# Five Breakthroughs: A First Approximation of Brain Evolution From Early Bilaterians to Humans

**DOI:** 10.3389/fnana.2021.693346

**Published:** 2021-08-17

**Authors:** Max S. Bennett

**Affiliations:** Independent Researcher, New York, NY, United States

**Keywords:** evolutionary neuroscience, evolutionary neuroanatomy, vertebrate brain evolution, mammalian brain evolution, primate brain evolution, bilaterian brain, brain evolution

## Abstract

Retracing the evolutionary steps by which human brains evolved can offer insights into the underlying mechanisms of human brain function as well as the phylogenetic origin of various features of human behavior. To this end, this article presents a model for interpreting the physical and behavioral modifications throughout major milestones in human brain evolution. This model introduces the concept of a “breakthrough” as a useful tool for interpreting suites of brain modifications and the various adaptive behaviors these modifications enabled. This offers a unique view into the ordered steps by which human brains evolved and suggests several unique hypotheses on the mechanisms of human brain function.

## Introduction

Humans have an incredibly diverse suite of intellectual faculties as well as incredibly complicated brains. But all these varied faculties and brain structures are likely to have evolved from simpler prototypes in the simpler brains of our ancestors. This general idea of progressive complexification of behavior and brains from simpler roots has been elegantly articulated in Paul Cisek’s theory of “phylogenetic refinement,” whereby behaviors and brain structures are interpreted as the consequence of evolutionary refinement from more basic building blocks (Cisek, 2019). An essential aspect of this research paradigm is chronicling the specific brain modifications that occurred in the human lineage, and what specific behavioral modifications they enabled.

Much work has been done to chronicle the specific brain modifications that occurred along major milestones in the human lineage from early bilaterians to extant humans (Kaas, 2009; Striedter and Northcutt, 2020). Work has also been done to reconstruct the adaptive *behavioral* *abilities* that emerged along major milestones in the human lineage from early bilaterians to extant humans (Murray et al., 2017; Cisek, 2019; Ginsburg and Jablonka, 2019; Bennett, 2021). A challenge of this research paradigm is how numerous the brain and behavioral modifications have been throughout evolution. The aim of this article is to introduce the concept of a “breakthrough” and use this concept to offer a first approximation explanation of a multitude of both brain and behavioral modifications that occurred during major evolutionary milestones. The general structure of the argument herein is that at each major milestone in human brain evolution, *many* neural modifications can be explained as having enabled a new *single* breakthrough, which was thereby applied in *many* adaptive ways.

### An Analogy to Technological Innovation: The Useful Concept of a “Breakthrough”

To elaborate on this idea, I will draw on an analogy to technological innovation. Consider the modifications and applications of a technological breakthrough such as the transition from gas-powered to electrically-powered households during the late 19th century. The *physical* *modifications* within a household were many: cables were laid, switches were added, circuit boards were installed. But all these new physical modifications can be reasonably understood through the lens of a single new *breakthrough* *capability* that they enabled—namely the capability of using electricity for energy. And this breakthrough capability was thereby *applied* in many adaptive ways: lighting a home at night for reading, heating a house during the cold without fire, speaking to faraway family members (using a telephone). The *value* of the physical modifications is defined only in the context of these adaptive applications.

Now imagine you were an alien observer trying to “explain” the observed physical changes to the 19th century home, as well as the observed new adaptive abilities of 19th century homeowners. Without a notion of the underlying breakthrough (electricity), and instead only with a model of the adaptive abilities and the physical modifications themselves, explaining the transformation of the 19th century home would be more perplexing. If you tried to explain individual physical modifications through the adaptive value they provided, it would be unclear: what was the specific adaptive value of a *single light switch* or a *single wire*? Conversely, if you tried to find the “substrates” underlying a given new “ability,” such as reading at night, the answer would also be unclear: entire suites of physical modifications worked together to enable these abilities. There was no single substrate. Further, many of the substrates of seemingly completely different newfound abilities are highly overlapping: reading at night, heating your home during the cold, and communicating at a distance all used overlapping physical features within a home (all used common cords, switches, and circuit breakers). In contrast, armed with the concept of a breakthrough, both the new physical modifications and the seemingly different new abilities have a much more interpretable first approximation: the varied physical modifications *together* served the single purpose of using electricity for energy, and this single breakthrough was applied in many different ways, including reading at night and communicating at a distance.

An additional benefit of the concept of a breakthrough is that it incorporates prior constraints into subsequent physical modifications and newfound abilities. Consider a technological breakthrough that occurred after that of electricity: the television (TV). Can we understand the modifications and breakthroughs of TV without first understanding that of electricity? TV was only possible because electricity came first and the ways in which electricity worked imposed constraints on how TVs would have to work. For example, TVs had to work on the relatively low voltage available within the home at the time. As such, we can only “explain” the specific modifications of TVs when we understand the constraints of the breakthrough that came before.

**Figure 1:**
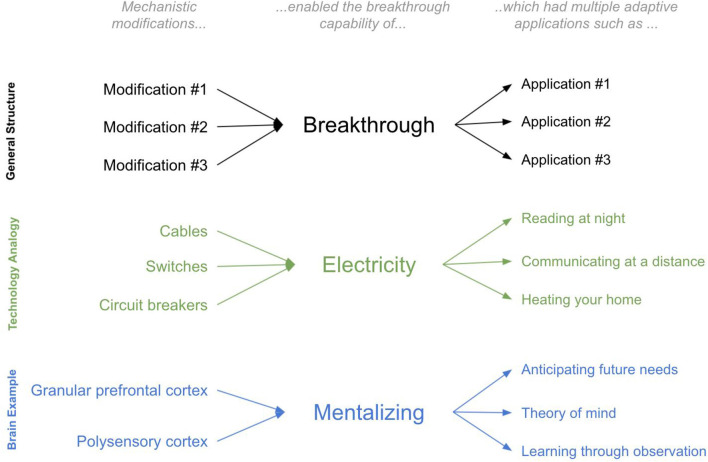
The concept of a “Breakthrough”.

In sum, there are two useful benefits of the concept of a breakthrough. The first is that it enables a useful first approximation of both physical modifications and the adaptive value they provide. The second is it provides a simple interpretation of ordered constraints, whereby prior breakthroughs imposed important constraints on future breakthroughs. The analogy is far from perfect. For example, numerous technological modifications to a home can be planned in advance, whereas evolution can only work via tinkering over each evolutionary iteration. Regardless, this analogy is illustrative—it offers an instructive lens for the concept of a breakthrough and the useful benefits it offers in explaining transformations (see Figure 1). This article is an attempt to apply this concept of ordered breakthroughs to brain evolution in order to offer explanations of the major brain and behavioral modifications throughout the evolutionary lineage from bilaterians to extant humans.

### The Approach

In the context of brain evolution, I will define three key terms:

- Physical Modifications: the actual physical changes in underlying neural structures.
- Breakthrough: a new capability that these numerous physical changes enabled, and which had numerous adaptive behavioral applications.
- Adaptive Applications: a new behavioral ability that offered survival and/or reproductive benefit to an animal and is one of many applications of an underlying breakthrough capability.

Below I will chronicle what I propose are the five major breakthroughs that occurred from the first bilaterians to the first human brains. There are undeniably many more modifications than will be described below, however, I argue that a remarkably broad set of brain functions and behaviors across taxa throughout the human lineage can be understood through the lens of only these five major modifications. I will connect these major modifications to likely behavioral abilities that emerged throughout our evolutionary timelines. This simplified model of brain evolution provides a useful “first approximation” of how and why brains evolved.

I intentionally call this a “*model*” and not a “*theory*.” I make the following distinction between the two: a model is a useful approximation of a set of phenomena, whereas a theory is a comprehensive explanation of a set of phenomena (Wunsch, 1994). Through this lens then, this article does not present a theory, as it is undeniably a simplification of the process of brain evolution. Instead, what it presents is a model—a useful first approximation that can be used to interpret and explain a multitude of observations.

Further, the scope of this article is intentionally anthropocentric—it seeks to chronicle the phylogenetic history of behavioral abilities in the *human* lineage between early bilaterians and extant humans. This requires an essential caveat to the hypotheses presented herein. Proposing a hypothesis regarding the emergence of abilities along the evolutionary lineage from early bilaterians to humans is not the same thing as proposing a hypothesis regarding a unique ability of humans relative to other extant animals alive today. For example, the hypothesis that episodic memory emerged in early mammals is not the same as a hypothesis that *only mammals* exhibit episodic memory. Convergent evolution is not the exception, but the rule.

I will start by presenting the model and then provide evidence for the model.

## The Model

### Breakthrough #1: Steering in Early Bilaterians

The hypothesis here is that the major neural modifications that emerged in early bilaterians facilitated the breakthrough of “steering,” which was thereby applied in multiple adaptive ways, such as in local area restricted search, avoiding predation, and maintaining homeostasis.

By “steering” I refer to the capability of categorizing external stimuli into two simple groups-positive valence (for approach) and negative valence (for escape). Agents can then turn towards directions where “positive valence” stimuli increase in potency (or “negative valence” stimuli decrease) and turn away from directions where “positive valence” stimuli *decrease* in potency (or “negative valence” stimuli *increase*). It is a breakthrough in the sense that an agent can navigate a complex environment remarkably well with only these simple categorizations and turning decisions. This navigational strategy is often called “taxis navigation,” whereby organisms move towards or away from specific stimuli and thereby climb up or down sensory gradients. Taxis navigation is present in single-celled organisms and almost definitely existed far before the first bilaterians. However, this model proposes that it was only in the first bilaterian animals where taxis navigation was implemented with neurons and muscles, and this unique implementation offered several additional benefits.

This model proposes that there were four physical modifications in early bilaterians: a bilateral body plan, valence sensory neurons that connected to global neural integration centers (the first “brain”), neurobiological mechanisms of associative plasticity, and neuromodulatory systems that generated persistent behavioral states. An interpretation of how these neural structures together implemented “steering” is as follows. A bilateral body plan reduced navigational decision making to simply forward or backward, and left or right (Holló and Novák, 2012). The sensory neurons of early bilaterians were evolutionarily hardwired to categorize specific external stimuli into those for “approaching” (forward) and others for “avoiding” (turn). These sensory neurons were directly sensitive to internal states. A chemosensory neuron in early Bilateria that was responsive to food cues would then also be directly modulated by hunger signals. In this way, the sensory apparatus of early bilaterians would have directly computed valence. Further, the global neural integration centers of early bilaterians would have been used to integrate competing input across valence neurons, which would have enabled the selection of a single cross-model decision in the presence of tradeoffs. For example, if an early bilaterian animal detected both the increase in a food cue (positive valence) and an increase in heat (negative valence), these different valence neurons would project to common interneurons where they could be integrated into a single decision of forward locomotion or turning depending on the relative strength of each valence response.

The mechanisms of associative plasticity, both postsynaptic and presynaptic, enabled early bilaterians to change the weights and hence valence of various stimuli. For example, experiencing pain in the presence of light could make light more aversive in the future. This enabled smarter steering decisions over time.

This model proposes that it was in these early bilaterians in whom neuromodulators such as dopamine and norepinephrine (and octopamine) were first used for valence-related signaling. Dopamine was released in the presence of food cues and persisted in extra synaptic space even after food cues disappeared. This enabled an animal to perform local area restricted search even in the absence of any specific food cues. Octopamine and norepinephrine did the same for negative valence stimuli, driving an animal to continue its relocation locomotion even after negative stimuli faded.

Together, these physical modifications enabled the breakthrough of “steering,” which enabled early bilaterians to optimally explore and exploit food patches while maintaining homeostasis. This implementation of taxis-navigation within *neurons* and *muscles* may have enabled comparably larger organisms to use taxis navigation than the prior taxis mechanisms of cellular cilia. Further, such a neuron implementation may have enabled more accurate sensitivity to internal states and cross-modal integration. This breakthrough was only possible because bilaterian brains were built on the foundation of neurons and muscles that evolved prior in eumetazoans.

### Breakthrough #2: Reinforcing in Early Vertebrates

The hypothesis here is that the brain modifications that emerged in early vertebrates facilitated the singular breakthrough of “reinforcing,” which was thereby applied in multiple adaptive ways, such as in map-based navigation, interval timing, and omission learning.

By “reinforcing” I refer to “model-free reinforcement learning”. In reinforcement learning, a distinction is often made between “model-free” and “model-based” methods. In simple terms, model-based methods include the ability to “plan,” which requires an agent to “play out actions” before taking them and choosing the sequence of actions that has the best outcome. This “playing out” of actions thereby requires a “model” of state-transition probabilities. In contrast, model-free methods include only learning the direct association with the *current* state and the available actions. The hypothesis here is that this “model-free” method of learning first emerged in early vertebrates, while the “model-based” method emerged later with the first mammals.

Model-free reinforcement learning requires several features: recognition of states, predicting the magnitude reward, predicting the *timing* of reward, temporal difference error signal, and the use of this error signal to update reward predictions. Despite these shared features, there are still many different implementations and conceptualizations of model-free reinforcement learning and how it manifests in brains. As such, two clarifications must be made regarding the features of model-free reinforcement learning this hypothesis proposes emerged in early vertebrates.

**Figure 2:**
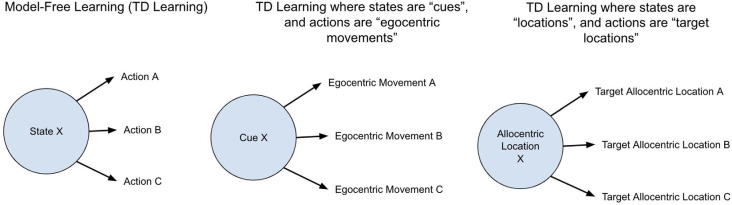
Different implementations of decision making in model-free reinforcement learning. The general structure of model-free reinforcement learning is agents learn to predict the discounted reward of each given state and learn what actions tend to increase the expected discounted reward from each given state. The definitions of an “action” and “state” can be implemented differently. Most interpretations consider a “state” as a cue and an “action” as a specific egocentric movement. The proposal here is that the model-free learning of early vertebrates was such that “states” were allocentric locations, and “actions” were *next target* allocentric locations. This enabled model-free learning that was flexible to slight differences in starting locations and self-correcting to mistakes such as overshooting a goal location.

The first clarification is regarding spatial maps. Model-based RL and spatial maps (sometimes called cognitive maps) are two concepts that sometimes get conflated—it has been suggested that evidence for the presence of a spatial map in an animal, as evaluated by various map-based navigational tests, is evidence for the presence of *model-based* reinforcement learning. The reasoning being that a spatial map requires a “model” of space. However, what makes an agent’s learning method *model-based* is not the *presence* of a spatial map but the *use* of that spatial map for the purpose of simulating future actions. A spatial map can still be used in the context of model-free learning without such simulation of future actions. For example, an agent’s current state can be defined as a location in space, and the actions it associates rewards with can be defined by the *next target locations*, which thereby would generate a homing vector from the current location to the next target location. This contains no “playing out” of state transition probabilities, but *does* have various adaptive properties, such as being robust to small changes in starting locations or paths (such as due to perturbations in water current). The hypothesis here proposes that spatial maps first emerged in vertebrates but they were *not* used for simulating future actions, and only used for learning associations between the current location and rewarding next target locations (see Figure 2).

The second clarification is regarding goal-directed vs. habitual behavior. Model-based learning and the ability to use reward *identity* in learning are sometimes conflated: it has been suggested that the presence of successful devaluation is evidence for the presence of model-based reinforcement learning. I will argue that this is not the case. A common experimental setup for such devaluation experiments is to allow a mouse to associate two levers each with a different type of food. Once this association is well learned, mice are allowed to eat one of those foods to satiation. When subsequently presented with each of these levers, mice will usually immediately favor the lever that produces the food that *was not* eaten to satiation (i.e., not devalued). Different experimental setups, as well as lesions of specific regions, can make mice insensitive to devaluation, whereby they will continue pushing the lever for the devalued food. Such behavior is typically considered to be “habitual” whereas that which is sensitive to devaluation is considered “goal-directed”. Habitual behavior at times is conflated with model-free learning: habitual behavior demonstrates direct stimulus-response associations, which can seem analogous to direct learning of rewarding actions from a given state in model-free learning. And similarly, goal-directed behavior clearly requires a “stimulus-stimulus” association, where each lever is associated with the food itself, and not just the original reward—this has been suggested to be evidence of model-based learning. However, neither is necessarily the case. If “states” include interoceptive information such as hunger level, then model-free learning can exhibit behavior that is sensitive to devaluation without any planning or playing out of future actions. Therefore, the proposal that early vertebrates were capable of model-free RL but *not* model-based RL does not suggest that all behavior of early vertebrates was habitual and insensitive to devaluation.

**Figure 3:**
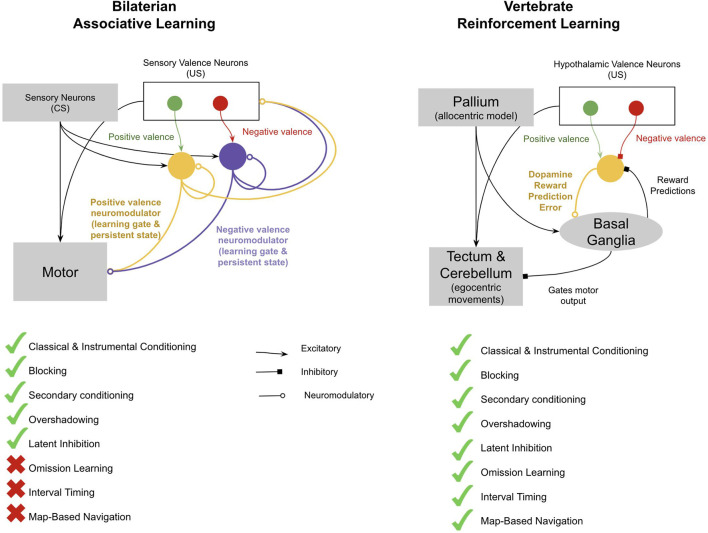
Proposed difference between the associative learning of early bilaterians and the reinforcement learning of early vertebrates. Invertebrates implement associative learning through accommodating neuromodulators, each for different valence and reflexes. This has many of the standard features of associative learning, such as blocking, secondary conditioning, overshadowing, and latent inhibition. However, it does not enable omission learning, interval timing, or map-based navigation. In contrast, the vertebrate reinforcement learning system has all these features. Key differences include the presence of a spatial map in the pallium; timing in the cerebellum, pallium, and basal ganglia (BG); and the fact that dopamine is used to encode both positive and negative reward prediction errors. See text for more details.

The main four new brain structures that emerged with early vertebrates were the pallium, the basal ganglia (BG), the tectum, and the cerebellum (Sugahara et al., 2017). This entire network of new brain structures of early vertebrates can be reasonably understood through the lens of the emergence of model-free reinforcement learning with spatial maps and timing. An interpretation of how these neural structures together implemented model-free RL is as follows (see Figure 3). Specific subregions of the pallium acquired the ability to represent allocentric representations of space (a “spatial map”), while others acquired the ability to recognize patterns of stimulus cues. Valence neurons in the evolutionarily older hypothalamus (inherited from the valence neurons of early bilaterians) became direct controllers of dopamine responses, whereby positive valence neurons *stimulated* dopamine and negative valence neurons *inhibited* dopamine. Activation of dopamine allowed long–term potentiation in the synapses between the pallium and the striatum of the BG. The BG then chunked together sequences of stimuli and places that tended to activate dopamine. The BG used these sequences to predict its own dopamine activations and used these predictions to *inhibit* dopamine neurons. This filtered out *expected* dopamine activations, thereby converting dopamine signals from a global valence signal to a *reward prediction error* (also called a “temporal difference learning signal”), which is an essential feature of model-free learning. The BG disinhibited neurons in the tectum, where allocentric representations from the pallium could be converted to egocentric movements, and hence drove movement towards specific “goal” locations activated in the BG. The pallial-BG system thereby learned sequences of allocentric goals that progressively climbed a dopamine gradient. This network generates “homing vectors” towards sequences of goal locations which are defined merely by climbing a dopamine gradient. Timing signals within the cerebellum, and potentially also in the pallium, enabled a representation of time that allowed animals to make choices not only based on *where* they are relative to an outcome, but also *when* they are relative to an outcome.

The breakthrough of model-free reinforcement learning would have offered early vertebrates numerous adaptive behavioral abilities. It would have enabled early vertebrates to navigate their environment not only with taxis-navigation, but also with map-based navigation—able to remember and navigate towards or away from specific locations in three-dimensional space. It would have enabled vertebrates to remember the specific timing between events, and thereby learn *when* to act. And it also would have allowed early vertebrates to not only learn from the presence of stimuli but also from the *omission* of stimuli. Such omission learning enabled vertebrates to perform much better on avoidance tasks.

Crucially, model-free learning would have only been possible in vertebrates because of the prior existence of valence neurons and neuromodulatory signals that evolved earlier in Bilateria.

### Breakthrough #3: Simulating in Early Mammals

The hypothesis here is that the unique brain regions that emerged in early mammals facilitated the singular breakthrough of “simulating,” which was thereby applied in multiple adaptive ways, such as in vicarious trial and error (VTE), episodic memory, and counterfactual learning.

By “simulating” I simply refer to the ability to perform *model-based* reinforcement learning, whereby an animal can play out and *simulate* action sequences before taking an action. In early mammals, the dorsal pallium of the ancestral amniote transformed into the neocortex (Tosches et al., 2018). I propose that the unique capabilities offered by this new neocortex relative to the pallium were its ability to internally invoke simulated actions and stimuli. A mammal with such an ability can pause, simulate a reality, manipulate it, evaluate it, and then act accordingly. This ability can be applied in many ways, such as for VTE (simulating paths), episodic memory (simulating a past event), or counterfactual learning (simulating alternative choices).

An interpretation of how the neocortex enabled such model-based reinforcement learning is as follows. The neocortex seems to be made up of a repeated columnar microcircuit (the “neocortical column”; Mountcastle, 1978). There are many competing theories of the specific computations performed by this microcircuit—including predictive coding (Rao and Ballard, 1999; Bastos et al., 2012; Spratling, 2017; Keller and Mrsic-Flogel, 2018), adaptive resonance theory (Grossberg and Versace, 2008), and hierarchical temporal memory (George and Hawkins, 2009; Hawkins and Ahmad, 2016; Bennett, 2020). Despite differences in these interpretations, they all generally agree that the microcircuit builds a self-supervised “model” with the purpose of predicting the entirety of its bottoms-up input. The self-supervised nature of the neocortex shares many features with a class of machine learning models called “generative models” (Kersten et al., 2004; Knill and Pouget, 2004; Parr and Friston, 2018). A generative model learns a “latent representation” (also called a “model” or an “explanation”) of its input. A generative model has two modes—an “inference mode” where it picks a latent representation that best “explains” its bottom-up input and a “generative mode” where it generates its own training data given a specific latent representation. Learning occurs by comparing the match between the simulated data and the actual data. Learning is optimized to minimize such mismatches (i.e., minimize “prediction errors”). Hence these generative models are “self-supervised”—trained only by the degree with which their own model of reality has successfully predicted its own input.

**Figure 4:**
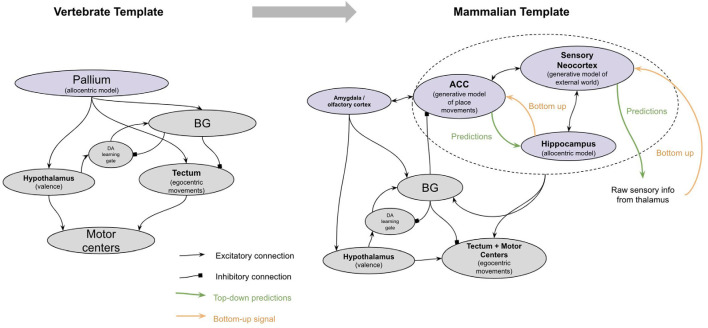
The major brain modifications in early mammals.

The neocortex of early mammals had two broad sub-regions: the frontal cortex and the sensory cortex (see Figure 4). The frontal cortex in early mammals is believed to be homologous to the anterior cingulate cortex (ACC) of later mammals (Laubach et al., 2018; van Heukelum et al., 2020). The sensory cortex had several homologous subregions for different modalities—visual areas, somatosensory areas, and auditory areas. This model suggests that each subregion implemented a generative model of sensor data from a given modality, with the goal of explaining its own input. However, this model proposes that the ACC served a different function, albeit performing an identical computation. Instead of receiving input from external sensory, the ACC of all mammals receives input from the amygdala and hippocampus (Reppucci and Petrovich, 2015), and projects throughout the sensory cortex (Zhang et al., 2014; Goll et al., 2015; Atlan et al., 2018; White et al., 2018; reviewed in Kamigaki, 2019). I hypothesize that the ACC is building a generative model of “paths” from the hippocampus, given “goals” from the amygdala. The goal represented is not a complex representation of the actual objects or sensory stimulus, but rather the actual valence results in the amygdala. The ACC thereby tries to explain the sequence of places that will be taken given a latent representation of a “goal” from the amygdala. One interpretation of this is that the latent representation in ACC is a model of “intent”—it observes an animal’s path, place, and context from the hippocampus and attempts to predict *why* the animal is behaving the way it is. This is consistent with other conceptualizations of generative models in the context of movement, often referred to as “active inference” (Adams et al., 2012).

**Figure 5:**
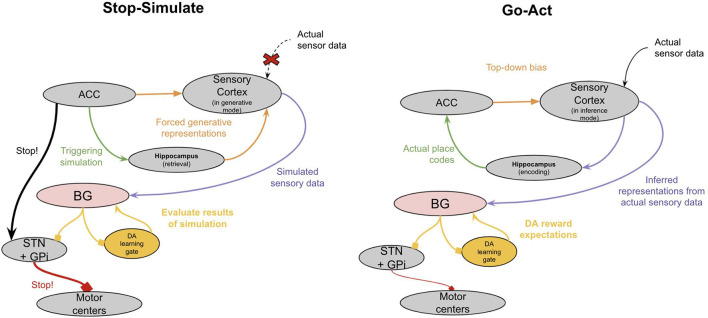
A high-level proposal of how “Simulating” is implemented in the neocortex. See text for details.

This model proposes that one function of this ACC representation of “intent” is its ability to trigger internally invoked simulations, which thereby allowed animals to engage in model-based learning (see Figure 5). When an animal reaches a “choice point” where the right answer is uncertain, this uncertainty is represented by multiple conflicting predictions from different columns of the ACC. This conflicting set of predictions triggers the animal to pause its movements. The neural substrate of this pausing may be the ACC direct projection to the subthalamic nucleus, which has been shown to be leveraged during top-down inhibition (Aron, 2006; Heikenfeld et al., 2020). The ACC can then trigger simulated paths through its loop with the hippocampus and can internally invoke the corresponding sensory representations of such paths through either its direct connections to the sensory cortex or through its indirect connections through the claustrum or hippocampus. During this “pause,” the generative model in the sensory neocortex shifts from being externally driven (“inference mode”) to internally driven (“generative mode”). The ACC will continue to explore “movements” consistent with its generative model of intent. These internally invoked representations of the world in the sensory neocortex can then be evaluated in the BG. When an imagined path finally achieves an outcome that leads the basal ganglia to release enough dopamine, it will trigger a “GO” response. This accomplishes two things. First, it immediately sensitizes the “imagined” path that the ACC triggered through the hippocampus, thereby biasing subsequent movements to be consistent with what was imagined. Second, it overcomes the ACC suppression of movement through the STN, enabling the evolutionarily older basal ganglia to take over behavior again.

During ongoing movement, when columns of ACC agree in their predictions of subsequent movement, the ACC can still exert some control over behavior by biasing the latent representations in the sensory neocortex to be consistent with the imagined path. This perhaps was the first version of “cognitive control” and “attention”. The ACC projects to sensory neocortex where it can perform “gain control” and bias sensory representations (Goll et al., 2015; Atlan et al., 2018; White et al., 2018).

Note that this “pause-simulate” behavior does not only apply to imagining action paths, but it also equally applies to imagining past events (episodic memory), imagining alternative choices to choices you already made (counterfactual learning), and perhaps even working memory (holding things “in mind”). The important feature is the ability of the ACC to trigger internally invoked simulations in the sensory cortex, which can be used to train the basal ganglia *vicariously*.

The motor cortex emerged in later mammals (placentals; Beck et al., 1996; Kaas, 2012). Motor cortex can also be interpreted through the lens of a frontal region that builds a model of “intent” and uses it to predict movement and trigger internally invoked simulation in the presence of uncertainty. In early mammals, the motor cortex was *not* required for moving in general but *was* required for movements that require preplanning. For example, movements where animals must grasp something they see or carefully step their feet on specific platforms requires simulating actions before moving—these fine movement skills are uniquely enabled by the motor cortex. The motor cortex can simulate these actions through its projections to the somatosensory cortex; the same way ACC can simulate paths through its projections to overall sensory cortex. The key difference between the motor cortex and the ACC is that the motor cortex gets its top-down input of “intent” from the ACC, and predicts specific body movements in the somatosensory cortex, while the ACC gets its top-down input of “intent” form the amygdala and predicts general navigational paths in the hippocampus. In this sense, the ACC relationship with the motor cortex was the first “motor hierarchy,” where goals flowed from the ACC to motor cortex. Therefore, the addition of the motor cortex can be viewed as an elaboration on the previous ACC-sensory network, which enabled the planning of fine motor movements.

The unique neocortical ability to trigger internally invoked simulations and use them for learning would not have been possible without two features inherited from earlier vertebrates. Firstly, spatial mapping in earlier vertebrates was repurposed in later mammals in order to explore environments vicariously. Without a spatial map, it would be impossible to simulate various movements and their consequences. Secondarily, internally invoked simulations work by training the basal ganglia *vicariously*—the basal ganglia does not have to tell the difference between an internally invoked or externally invoked sensory data from the sensory cortex, it merely learns what sequences of movements trigger dopamine release. This ability to learn *vicariously* was only possible because it was built on top of the foundation of the older basal ganglia.

### Breakthrough #4: Mentalizing in Early Primates

The hypothesis here is that the unique brain regions that emerged in early primates facilitated the singular breakthrough of “mentalizing,” which was thereby applied in multiple adaptive ways, such as in anticipating future needs, theory of mind, and learning skills through observation.

By “mentalizing” I refer to the ability to construct a model of the *mind*, inclusive of an individual’s *intent* and *knowledge*. Such a model of mind can be *applied* in multiple ways—three such ways are “anticipating future needs,” “theory of mind” and “learning skills through observation.” All three of these can be seen merely as different applications of this singular ability of primates to engage in “mentalizing.” For example, mentalizing can be used to simulate a mind state of *yourself* that you do not have yet (imagining being hungry tomorrow if I do not gather food now, even though I am not hungry right now). It can be used to simulate the mind state of *another* conspecific you are observing (such as imagining how they must feel given their situation). It can be used to simulate the *intentions* and *actions* of others when you are watching them do various motor skills, which enables you to “learn by observation.” The behavioral manifestations are different, but the neural substrates are overlapping for a reason: the overall function is the same (simulating a mind state).

**Figure 6:**
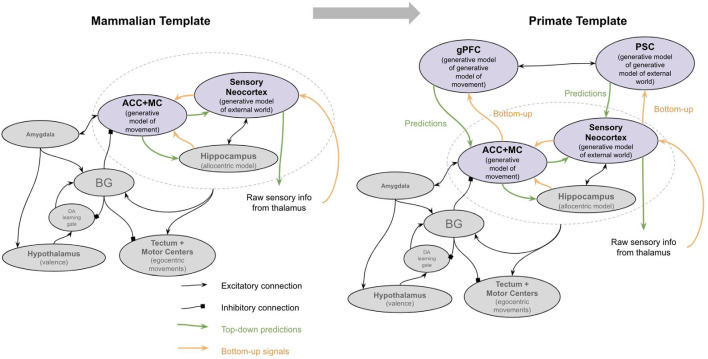
The major brain modifications in early primates. See text for details. PP, Posterior parietal cortex; MC, motor cortex; PSC, polysensory cortex (superior temporal cortex and temporoparietal junction); gPFC, granular prefrontal cortex; ACC, anterior cingulate cortex; BG, Basal ganglia.

The primary two new brain structures that emerged in early primates were the granular prefrontal cortex (gPFC) and polysensory cortex (PSC; Kaas, 2009). An interpretation of how granular prefrontal cortex and PSC enabled such mentalizing is as follows (see Figure 6). gPFC gets its inputs from three primary sources: (1) it is bidirectionally connected with the ACC, both directly and through the mediodorsal thalamus (Kondo et al., 2004; Cera et al., 2019; Tang et al., 2019); (2) gPFC is interconnected with polysensory areas such as the superior temporal cortex and TPJ (Sanfey, 2003; Greene et al., 2004; Buchsbaum et al., 2005; Tei et al., 2017); and (3) it is interconnected with motor cortex both directly and indirectly through descending loops through the motor thalamus (Bosch-Bouju et al., 2013; Yokoi and Diedrichsen, 2019). I hypothesize that gPFC and PSC together implement a generative model *of the ACC-sensory generative model itself*. In other words, the gPFC-PSC generative model is constructed to “explain” the “intentions” from the ACC given “knowledge” from PSC. The emergent property of this is that it is effectively a generative model of one’s own “mind,” the use of which can be thought of as “mentalizing”.

When applying this gPFC-PSC network to “mentalizing about *oneself*,” an animal can anticipate a need it does not currently feel yet. One big difference between the ACC-sensory network and the gPFC-PSC network is that the ACC-sensory network is self-supervised to predict amygdala and hypothalamic activation, while the gPFC-PSC is self-supervised to predict latent representations within ACC-sensory network. The ACC-sensory network can predict paths that accomplish the needs currently experienced. On the other hand, gPFC-PSC network can simulate situations in the future and predict what intentions *would* be selected in the ACC-sensory network *given* such situations. The ACC-sensory network can’t do this because it is supervised to predict amygdala activation, which is therefore only sensitive to *current needs*. One reason this new network was adaptive was because it enabled early primates to anticipate future needs and hence plan much more flexibly than the mammals that came before. Practically, this enables primates such as humans to go to the grocery store and pick up food for the week even when they are not yet hungry—or to bring a jacket on a trip even though they are not yet cold. This ability to “anticipate future needs” was originally thought to be unique to humans, summarized as the Bischof-Kohler Hypothesis (Bischof-Köhler, 1985), but this model proposes that it in fact evolved in early primates, which as we will see in later sections is consistent with more recent tests in nonhuman primates and non-primate mammals.

Once a generative model of one’s own mind exists, it can be applied not only to “mentalizing about *oneself*” but also to “mentalizing about *others*”. Because the gPFC-PSC network is a model of what behaviors are generated from what intentions and knowledge, it can be applied to trying to identify what intentions and knowledge in others *are consistent with their observed behaviors*. The same way that the ACC-sensory system pauses and simulates objects and paths, the gPFC-PSC system can pause and simulate intentions and knowledge of others to generate a latent representation most consistent with the observed behavior. This mentalizing about others can manifest itself in several ways. First, it can be used in the basic theory of mind tasks to infer the knowledge of others given their perspective. And second, it can be used to learn motor skills through observation.

One attractive feature of this proposal of the gPFC-PSC network is that it fits within the conceptualization of the neocortical microcircuit. Given the observation that the neocortical column seems remarkably uniform throughout the neocortex (Mountcastle, 1978), any purported new “function” attributed to a new neocortical region should be primarily a consequence of unique inputs and outputs, as opposed to any changes in the underlying computations of the neocortical column. Consistent with this, the idea here is that the microcircuitry of the granular prefrontal cortex is the same as that of the ACC and sensory cortex: it is still implementing a generative model. The difference is merely where it receives input from. The ACC constructs a model getting bottom-up input from the hippocampus. The latent representation in the ACC is then a representation of an animal’s *current* intent (intent is an “explanation” of an observed path), which is used to make predictions of subsequent navigational paths. In contrast, the granular prefrontal cortex gets bottom-up input from the ACC. The latent representation in the granular prefrontal cortex is then a representation of a mind state (mind state is an “explanation of an intent”) which is then used to construct make predictions about one’s own or others’ *intent*.

Crucially, simulating mind states (mentalizing) in early primates was only possible because of the inherited ability to *simulate world states* that evolved in earlier mammals. Mentalizing was built on the foundation of simulating world states. The model here proposes that mentalizing network itself is a model of the mammalian ACC-sensory network, literally built on top of the evolutionarily older brain structures.

### Breakthrough #5: Speaking in Early Humans

The hypothesis here is that the unique brain regions that emerged in early humans facilitated the singular breakthrough of “speaking,” which was thereby applied for language and music.

By “speaking” I do not refer only to vocal communication, but broadly to semantic rhythmic communication in general. Human brains, although bigger, are remarkably similar to the primate brains that came before. However, one fundamental difference that seemed to emerge was a modification to the arcuate fasciculus and its connectivity with the basal ganglia (“AF-BG network”). I hypothesize, as have others, that the neural innovations for language and music emerge from the unique connectivity of the arcuate fasciculus, as well as perhaps other additional connectivity with the striatum (Fujii et al., 2016). This has been the classic view of language since the time of Wernicke (Wernicke, 1874; Stookey, 1963; Berker et al., 1986; Anderson et al., 1999). More modern theories of language have criticized the simplicity of this original model and proposed additional structures (Rasmussen and Milner, 1977; Imaizumi et al., 1997; Rauschecker and Scott, 2009; Chang et al., 2015). I do not make any specific claim on the exact mechanisms of language production, merely that language and music were emergent properties only possible with the additional connectivity of the arcuate fasciculus.

The view here is that music and language are two sides of the same coin and emerge from the same neural innovations. There is an intuitive appeal to this hypothesis, as both music and language share many features. Both require rhythmic entertainment coordinated with other conspecifics. In other words, “beat perception” is necessary for both music and for taking turns appropriately in a conversation. Both are hierarchical and nested (Drake et al., 2000; Toiviainen and Snyder, 2003; McKinney and Moelants, 2006; Martens, 2011)—music contains beats within “bars” within “phrases,” and language contains phonemes within words within sentences. Both are highly “predictive”—when you hear an unfinished sentence such as “the ford mustang is my favorite …,” you cannot help but finish it. This is the same for music, when you hear an unresolved musical phrase.

In the context of this model, one explanation for the ordering of these breakthroughs is that such rhythmic semantic processing for communication was only possible *after* the breakthrough of “mentalizing”. Only with the ability to infer and understand the knowledge of others is one able to devise a reasonable communication to transfer information to someone. Consistent with this, the AF-BG network is very overlapping with the mentalizing regions that came before.

### Model Summary

**Figure 7:**
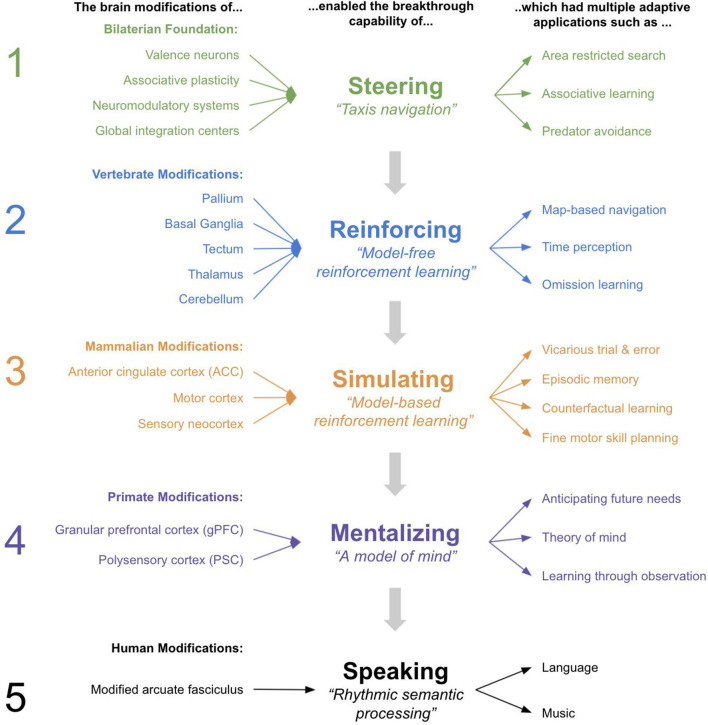
Summary of the 5 Breakthroughs from early bilaterians to humans.

In Figure 7, you can see a summary of the proposed five breakthroughs, and the modifications and behaviors they explain. You can also see the homologous regions in human brains.

As previously mentioned, one attractive feature of this model of five breakthroughs is that it incorporates evolutionary constraints and thereby helps understand how neural innovations build on each other. Consider the following dependencies suggested by this model.

Steering in early bilaterians was only possible because of the neural building blocks that emerged earlier in eumetazoans, which provided all-or-nothing neurons with sensory cells and muscle cells. It provided inhibitory and excitatory neurons for the creation of neural circuits, and neuropeptides for the modulation of neural responses based on internal states.

Reinforcing (model-free reinforcement learning) in early vertebrates was only possible because of the features of steering in the ancestral bilaterian brain. The core mechanism for learning sequences of places is the temporal-difference learning mechanism which bootstraps these learned responses on hardcoded valence responses. Ancestral bilaterians had such valence responses that could stimulate the release of various neuromodulators. The pallial-BG-tectal circuit could then build plastic networks on top of these basic valence responses to enable learning more complex sequences of paths. This is consistent with other interpretations of behavior as evolving (through phylogeny and ontogeny) from the basic foundation of approach and withdrawal (Schneirla, 1959).

Simulating (model-based reinforcement learning) in early mammals was only possible because of model-free reinforcement learning in ancestral vertebrate brains. Simulating requires: (1) a “pointer” to a specific suite of stimuli; and (2) a mechanism for manipulating key features of the stimuli once invoked. The spatial map in the pallial hippocampus provided both of these features—internally invoking a “place” in the pallial hippocampus will reactivate a broad suite of learned responses to that place. Further, the ability to tether different places together and generate novel paths, enables the pallial hippocampus, if it were internally invoked, to imagine “novel” paths. It is then no surprise that the neocortex is, in some ways, built directly on top of the much older hippocampus, and uses the hippocampus for its simulating functions, such as VTE and episodic memory.

Mentalizing in early primates was only possible because simulating in ancestral mammal brains came first. In the model described here, mentalizing is computationally the same as simulating, the only difference being that mentalizing uses a generative model of the mind itself, requiring new neocortical regions and new connectivity between them. Mentalizing could only occur after there was already a generative model of intent on which to simulate possible mind states.

And lastly, language and music in early humans were only possible because of mentalizing in ancestral primate brains. There are several pieces of evidence for this. First, language and music are built directly on top of the neural structures of mentalizing: they emerge from uniquely primate mentalizing areas such as STS, TPJ, and the prefrontal cortex. Second, mirror neurons have been highly implicated in both theories of language origin as well as language function (Gallese et al., 1996; Rizzolatti et al., 1996; Rizzolatti and Arbib, 1998; Fogassi et al., 2005; Schooler et al., 2011; Vredeveldt et al., 2011; Yonemitsu et al., 2018). Third, people with disorders that disrupt mentalizing abilities, such as autists, also show language impairments (Mitchell et al., 2006; Luyster et al., 2008; Mody and Belliveau, 2013). And lastly, as an infant develops, the advanced mentalizing abilities emerge first in children before language emerges, with “intersubjectivity” and “joint attention” occurring before words (Bateson, 1975; Beebe et al., 1988; Carpenter et al., 1998; Meltzoff and Brooks, 2001; Bard et al., 2005). These “theory of mind” abilities are a requirement for the development of language abilities (de Villiers, 2007). The connection between mentalizing and language makes sense through the lens of various theories of the evolution of language. If the original evolutionary pressure for language was in fact for communicating the type and location of faraway food to conspecifics, as has been proposed (Monahan, 1996), then it makes sense that it would at first require mentalizing: the goal of the communication is to figure out what your communication partner does not know that you want them to know, and how to use shared understanding to transfer this knowledge.

## Evidence for the Model

Three categories of evidence are presented in support of the model presented here. First, evidence is presented to support the proposed phylogenetic timing of specific brain modifications. Second, evidence is presented to support the proposed phylogenetic timing of specific behavioral abilities. And third, evidence is presented to support the proposed *function* of these brain modifications and how they enabled the specific behavioral abilities. For brevity, much of the evidence presented will direct readers to other work where the evidence for various claims is presented more thoroughly. Some of the claims are less controversial than others; extra attention will be spent on the claims for which there is more controversy.

### Evidence For the Proposed Phylogenetic Timing of Brain Modifications

#### Evidence That a Global Neural Integration Center (The First “Brain”) Emerged in Early Bilaterians

The prevailing view is that the first neurons and nervous systems emerged in the common ancestor of eumetazoans (“real metazoans”) during the Ediacaran period around 600 million years ago (Peterson and Butterfield, 2005). Animals before Eumetazoa are believed to be devoid of nervous systems, akin to extant sea sponges (Bucher and Anderson, 2015). In these early Eumetazoans, nervous systems were not organized into any recognizable “brain,” but rather a diffuse “nerve net” (Holland, 2003; Lowe et al., 2003; Galliot and Quiquand, 2011; Arendt et al., 2015). Evidence for this comes from the nerve nets of cnidarians, which are some of the earliest diverging eumetazoans.

Despite a lack of a brain, nonbilaterian eumetazoans such as cnidarians have a surprisingly rich set of neural features, strongly suggesting that early eumetazoans already contained many of the building blocks of brains. For example, neurons in cnidarians communicate using “all-or-nothing” action potentials and form chemical synapses with each other (Satterlie, 2015). Cnidarian neurons contain many modern-day neurotransmitters, including glutamate, GABA, and acetylcholine (Kass-Simon and Pierobon, 2007; Marlow et al., 2009; Delgado et al., 2010; Pierobon, 2012). Cnidarians have also been shown to contain many of the same ionotropic and metabotropic receptors for glutamate (both AMPA and NMDA), GABA, acetylcholine, and even some monoamines (Anctil, 2009; Collin et al., 2013; Bosch et al., 2017).

Neural circuits within cnidarians, and hence also likely early eumetazoans, also came with several well-known features of neurons across taxa such as adaptation and interoception. Adaptation, whereby animals respond less to repeated stimuli, has been shown in various responses such as the tentacular responses in hydras and sea anemones (Parker, 1916; Pantin and Pantin, 1943; Batham and Pantin, 1950). Interoception, whereby animals modulate their responses based on internal cues for various need states, has also been shown in cnidaria. For example, hydras and sea anemones seem to be less sensitive to food cues (less likely to trigger the feeding response and less responsive to mechanical stimulation) when full than when hungry (Parker, 1916; Pantin, 1935; Batham and Pantin, 1950; Lenhoff and Loomis, 1963; Han et al., 2018).

In contrast, even very early diverging bilaterians, such as *C. elegans* and planarians, have global neural integration centers that can be thought of as a “brain” (Garrity et al., 2010). Although there is still controversy regarding whether the first bilaterians had a neural net or an actual brain (Hejnol and Martindale, 2008; Arendt et al., 2015), both interpretations agree that the first brain emerged within the bilaterian lineage.

#### Evidence That the Pallium, Basal Ganglia, Tectum, and Cerebellum Emerged in Early Vertebrates

Before the first vertebrates, brains are believed to have been made up of a homolog of a hypothalamus, midbrain, and hindbrain, as in early diverging chordates such as amphioxus (Nieuwenhuys, 1998; Gorbman et al., 1999; Uchida et al., 2003; Murakami et al., 2005). There is a general consensus that with vertebrates, at least five additional structures emerged: the pallium, the BG, the tectum, the cerebellum, and the thalamus (Sugahara et al., 2017). Evidence mostly comes from the fact that these structures, their microcircuits, and the canonical connectivity between them are highly shared amongst extant vertebrates. Even the lamprey, considered one of the earliest diverging vertebrates and considered a model organism for the early vertebrates, shares exactly this template (Grillner and Robertson, 2016). Evidence suggests the amygdala, hippocampus, and neocortex all evolved from the pallium: specific subregions of the pallium in early diverging non-mammal vertebrates such as fish seems to be a proto-hippocampus (Macphail, 1982; López et al., 2003), and the other subregions of the pallium a proto-amygdala or proto-olfactory cortex, performing similar functions and containing similar microcircuits.

Some protostomes, such as arthropods, have brain regions that are structurally and functionally similar to the vertebrate pallium and basal ganglia, such as the mushroom body (similar association properties as the pallium) and central complex (similar properties as the basal ganglia). Some have argued that these structures share homology with vertebrate structures, suggesting that precursors to them emerged early in Bilateria (Strausfeld and Hirth, 2013). However, others argue it is simply an example of convergent evolution (Northcutt, 2012; Farries, 2013). Given that these structures are entirely absent from most protostome taxa, including nematodes, flatworms, mollusks, and annelids, the principle of parsimony makes convergent evolution seem more likely. Further, modern views of the urbilaterian ancestor suggest that its brain was devoid of such structures and instead was made up of a simple sensory region and a motor region (Arendt et al., 2015). Either way, the protostome versions of these structures have many more differences from the vertebrate versions than the vertebrate versions have from each other. This implies that the modern form of these structures first emerged in early vertebrates.

#### Evidence That the Neocortex With ACC and Sensory Regions Emerged in Early Mammals

There is broad consensus that the six layered neocortex first evolved in early mammals, and was derived from the three layered dorsal pallium in earlier amniotes (Tosches et al., 2018). Along with this modification, the surrounding pallial structures are also believed to have become recognizable in their mammalian forms—the medial pallium became the mammalian form of the hippocampus, and the lateral pallium became the mammalian olfactory cortex and/or the amygdala (Kaas, 2009; Tosches et al., 2018). The basal ganglia, tectum, thalamus, hypothalamus, and other midbrain and hindbrain structures are remarkably similar across non-mammal vertebrates and mammals alike, strongly suggestive that they were left relatively unchanged in early mammals.

Although there is little controversy regarding the evolution of the neocortex in early mammals, the degree to which it is “different” from the structures that emerged earlier is somewhat controversial. For example, some evidence suggests that the dorsal ventricular ridge (DVR) of birds (containing the “nidopallium” and “mesopallium”) and the neocortex of mammals both derive from the pallium of their shared amniote ancestor (Karten, 1969, 1997; Reiner et al., 2004; Dugas-Ford et al., 2012). It has been shown that the DVR and neocortex share many features, including molecular properties and the subcortical structures they interact with. Hence, this might be used to call into Question the uniqueness of the mammalian neocortex, and perhaps suggests that the amniote common ancestor had neocortex-like structures. However, this interpretation is unconvincing for two reasons.

First, the brains of birds are a poor model organism for the amniote last common ancestor. It is not even clear that the DVR itself is homologous with the neocortex and instead might share homology with the mammalian amygdaloid complex (Jarvis et al., 2005; Striedter, 2005). Further, even if the DVR does share homology with the neocortex, the microcircuitry of the DVR is very different from that of the neocortex. The neocortex is organized into six layers, while the DVR is organized into clustered nuclei (Ulinski, 1983). The ontogeny of the DVR and the mammalian neocortex is different (Jones and Levi-Montalcini, 1958; Striedter and Keefer, 2000; Dugas-Ford et al., 2012). Additionally, the pallial homologs in non-bird and non-mammal extant amniotes such as reptiles, also have unique ontogeny and microcircuitry from both birds and mammals (Goffinet et al., 1986; Cheung et al., 2007). For example, turtles have a three layered pallium, instead of the clustered nuclei of the DVR or the six layered neocortex. The turtle cortex is more like the three layered pallium of other non-amniote vertebrates, such as fish (Mueller and Wullimann, 2009), than it is to the DVR of birds or the neocortex of mammals. This is more consistent with the proposal that early vertebrates had a three layered pallium, much like that of extant reptiles and teleosts, and that the pallial homologs in birds and mammals each underwent substantial independent modification since the amniote last common ancestor.

Although there is considerable debate regarding which neocortical regions emerged first, it is generally accepted that by the time the early placental mammals emerged around 65 million years ago the following neocortical areas existed: V1, S1, A1, M1, cingulate cortex, insular cortex, orbital frontal cortex (Kaas, 2012). Even in earlier diverging mammals such as marsupials, which diverged from our lineage over 150 million years ago, there are numerous neocortical areas, such as a cingulate, V1, S1, and A1 (Karlen and Krubitzer, 2007; Wong and Kaas, 2009). This is consistent with the model herein which proposes that the very early neocortex contained both frontal regions and sensory regions. I use the label ACC to refer to the entirety of the agranular frontal cortex in early diverging mammals, inclusive of the areas sometimes called prelimbic or infralimbic cortex. Consistent with this, evidence suggests the entire prefrontal cortex of rodents is homologous to the anterior cingulate of primates (Laubach et al., 2018; van Heukelum et al., 2020).

#### Evidence That the Major Brain Modification of Early Primates Was the Addition of the “gPFC-PSC Network”

Primate brains are bigger in size than earlier diverging mammals relative to the overall body, but for the most part, contain the same neural structures. Some relative differences within primate brains include a shrinking of the olfactory bulbs and a substantial expansion of the visual cortex, somatosensory cortex, and posterior parietal cortex. However, the four substantial differences in the brains of extant primates from earlier diverging mammals are: the addition of (1) granular prefrontal cortex (gPFC; premotor areas, dorsolateral prefrontal cortex, and frontopolar cortex; Semendeferi et al., 2001); (2) PSC (including the superior temporal sulcus and the temporoparietal junction); (3) the dorsal pulvinar (Preuss, 2006); and (4) a unique cortico-motoneuronal system, where corticospinal projections bypass older circuits and make direct connections with spinal motorneurons (Murabe et al., 2018; Lemon, 2019). Although some suggest such direct corticospinal projections also occur in rats (Elger et al., 1977; Carlin et al., 2000; Yang and Lemon, 2003; Alstermark et al., 2004; Maeda et al., 2015; Gu et al., 2017), new evidence shows that these direct projections disappear in adulthood (Murabe et al., 2018), unlike in primates (Armand et al., 1997; Eyre, 2007).

The predominant view is that these structures and modifications emerged in early primates as they do not seem to exist in non-primate mammals, while they do exist in most extant primates (Kaas, 2009). Further consistent with the proposed model, and the idea that these new structures served a single purpose, the dlPFC, temporal cortex, and parietal areas all make up their own interconnected network, through the uniquely primate dorsal pulvinar (also called medial pulvinar; Goldman-Rakic, 1988; Gutierrez et al., 2000).

#### Evidence That the Major Brain Modification of Early Humans Was a Modification to the Arcuate Fasciculus

There are two main differences in the connectivity of human brains relative to those of non-human primates. First, the arcuate fasciculus (AF), which is a network of cortico-cortical connections between areas in the prefrontal cortex (“broca’s area”) and areas in the posterior cortex (“Wernick’s area”), is massively expanded in humans (Aboitiz and Garciía V, 1997; Aboitiz et al., 2006, 2010; Rilling et al., 2008, 2012; Aboitiz, 2012; Catani and Bambini, 2014; Petrides, 2014; Rilling, 2014; Stout and Hecht, 2017) and contains unique connectivity (Petrides and Pandya, 1984; Catani et al., 2004; Schmahmann et al., 2007; Rilling et al., 2008; Thiebaut de Schotten et al., 2012). Second, in humans, there is a direct projection from the motor cortex to laryngeal motor neurons, which is not found in nonhuman primates (Fitch, 2018; Jarvis, 2019). Although Broca’s area and Wernicke’s area are highly implicated in unique human abilities such as language, homologous regions of both have been found in nonhuman primates (Cantalupo and Hopkins, 2001; Schenker et al., 2009; Spocter et al., 2010), and therefore do not seem to have uniquely emerged in early humans.

### Evidence for Proposed Phylogenetic Timing of Behavioral Abilities

Elsewhere I have more thoroughly reviewed the evidence for the below hypotheses regarding the phylogenetic timing of behavioral abilities during brain evolution in the human lineage (Bennett, 2021). I will briefly review some of the evidence here.

#### Evidence That Taxis-Navigation and Associative Learning Emerged in Early Bilaterians

To clarify, the hypothesis here is that taxis navigation, implemented in neurons and muscles, first emerged in bilaterians, and not that taxis navigation in general first emerged in bilaterians. Because this article is interested in *brain* evolution, the functions and features of the first brains are the focus of this article. Therefore, taxis navigation that existed in non-neural substrates is not explored in detail. *C. elegans* and flatworms, generally considered model organisms for urbilateria, demonstrate clear taxis-based navigation (Pearl, 1903; Larsch et al., 2015). These model organisms are also able to perform cross-modal decision-making. They can integrate conflicting input from various modalities (thermosensation, mechanosensation, photosensation, and chemosensation) in order to make a single integrated navigational decision. For example, *C. elegans* will make different decisions about whether to cross a copper barrier (which is aversive) to get to the food on the other side depending on the strength of the food smell relative to the concentration of copper (Ishihara et al., 2002). Similarly, flatworms will navigate towards food despite an aversive light source, but as the light becomes brighter, they will not go as far towards the food. This type of integrated decision-making occurs across numerous modalities including mechanosensation and thermosensation (Inoue et al., 2015). The taxis navigation of these model organism also demonstrate persistent navigational states, which would have been useful for persisting navigational decisions even after sensory stimuli have faded. For example, the *C. elegans* demonstrates at least three behavioral states: roaming, dwelling, and sleep (Fujiwara et al., 2002). Roaming is categorized as primarily straight-line swimming with infrequent turns—enabling an animal to relocate. In contrast, dwelling is categorized by slow swimming and frequent turning, enabling an animal to “locally search” its general area.

On the other hand, evidence for taxis navigation in adult form non-bilaterian eumetazoans is sparse. The hunting strategy of sea anemones, believed to be a model organism for early cnidarians (Yuan et al., 2011), is to wait for food to come to them (Ruppert et al., 2004). Retraction reflexes in cnidarians don’t drive locomotion in a specific direction, and instead seem to simply globally increase arousal (Batham and Pantin, 1950). Even most medusae, a more complex adult cnidarian form that likely evolved after the cnidarian-bilaterian divergence, do not show taxis navigation towards food sources, and merely seem to orient themselves in direction of current (Fossette et al., 2015), and hunt by moving in a “levy walk” (Hays et al., 2011). There are admittedly some exceptions to this. Box jellyfish can use eye spots to avoid obstacles (Garm et al., 2007), but it is generally accepted that the eyes of box jellyfish evolved independently (Nilsson, 2013; Bosch et al., 2017). Sea anemones have been shown to move towards light sources (Parker, 1916), but this has been shown to be independent of their own visual apparatus and driven simply by nearby amoebae (Pearse, 1974; Foo et al., 2019).

While larvae of earlier diverging metazoans, such as sponges, show taxis navigation (Leys et al., 2002), their adult forms show no such behavior and the neural implementation is based on cilia and not neurons and muscles. Adult forms of ctenophores move through ciliated pumping which may be coordinated via neurons and therefore may represent an adult form metazoan with neuron-based taxis navigation. However, how well extant ctenophores represent early metazoans is unclear; much evidence favors the idea that ctenophores independently evolved many features of nervous systems (Ryan, 2014; Moroz, 2015; Moroz and Kohn, 2016; Liebeskind et al., 2017). This would suggest that ctenophore taxis navigation is not indicative of early metazoans before the cnidarian-bilaterian last common ancestor.

Most evidence also suggests that associative learning first emerged in early bilateria (Bennett, 2021; Ginsburg and Jablonka, 2021). Associative learning, including classical conditioning and instrumental conditioning, has been shown broadly across Bilateria, including even early diverging species such as aplysia (Hawkins et al., 1989), planarians (Prados et al., 2012), and *C. elegans* (Ardiel and Rankin, 2010). In contrast, attempts to find associative learning across cnidaria have shown primarily negative results (Rushforth, 1973; Torley, 2009). Admittedly there is a single report of associative learning in a sea anemone (Haralson, 1975)—however, this is inconsistent with most other studies. Similar to others, I conclude that cnidaria do not contain associative learning (Ginsburg and Jablonka, 2019), and at the very least, if they do, it evolved convergently.

It should be noted that there are different interpretations of associative learning. Some make a distinction between “alpha conditioning” and “beta conditioning” (Razran, 1971; Moore, 2004). “Alpha conditioning” is defined as that where a non-habituated CS elicits the same reflexive response as the US, but after pairing with the US the *magnitude* of the response elicited by the CS increases. In contrast, “beta conditioning” is considered “true associative learning” whereby the CS elicits no reflexive response before pairing. However, others disagree with this distinction (Hawkins and Kandel, 1984; Kandel, 2006), and instead argue that there is, in fact, no difference between alpha and beta conditioning—they argue that all wiring is pre-wired, the only difference is whether the wiring is strong enough to elicit a response before pairing. All these perspectives agree that there is a distinction between *general sensitization*, where the specific US globally sensitizes a suite of reflexive responses, and *associative learning*, whereby this sensitization is local and specific to the paired US.

#### Evidence That Map-Based Navigation, Interval Timing, and Omission Learning Emerged in Early Vertebrates

In the context of map-based navigation, diverse and early diverging vertebrates including fish (Burt de Perera et al., 2016), reptiles (Wilkinson and Huber, 2012; Broglio et al., 2015), turtles (López et al., 2001), amphibians (Phillips et al., 1995), and tortoises (Wilkinson et al., 2007) all show the ability to build spatial maps of their environment and flexibly generate novel navigational routes to known places (Rodríguez et al., 2002). Model organisms for early bilaterians, such as flatworms seem to navigate only with taxis and perhaps response-based learning (Pearl, 1903; Luersen et al., 2014; Larsch et al., 2015; Gourgou et al., 2021) and show no ability to remember specific un-cued locations. Further, the neural substrates of such map-based navigation in vertebrates are uniquely vertebrate structures, such as pallial homologs of the hippocampus, suggestive of vertebrate origins. Importantly, the map-based navigational tests that early diverging vertebrates, such as fish, pass do not require *planning*, but they do require a spatial map that can generate novel homing vectors to well-learned locations in three-dimensional space. There is indeed evidence of sophisticated 3-dimensional navigation in arthropods, however, the degree to which they truly represent map-based representations and the degree to which these abilities are representative of early bilaterians is unclear. Some evidence suggests that arthropods fail at map-based navigation tasks (Benhamou et al., 1990; Wehner et al., 1996, 2006; Walker, 1997), while other evidence suggests that they indeed can build map-like memories (Boles and Lohmann, 2003; Menzel et al., 2005, 2011). Further, many impressive abilities of arthropods emerge from mushroom bodies (Perry et al., 2013; Cope et al., 2018), which is a cortex-like structure believed to have evolved independently (Farris, 2008).

In the context of interval timing, diverse and early diverging vertebrates such as fish (Sumbre et al., 2008), birds (Bateson and Kacelnik, 1997; Ohyama et al., 1999; Buhusi et al., 2002), non-human primates (Gribova et al., 2002), and mice (Roberts and Church, 1978; Gallistel et al., 2004; Buhusi et al., 2005) all show the ability to remember the precise timing between two cues. In contrast, invertebrates show a weak perception of time, if one at all (reviewed in Abramson and Wells, 2018). Further, the neural substrates of interval timing in vertebrates seem to be uniquely vertebrate structures (Buhusi and Meck, 2005; Yin and Meck, 2014).

In the context of omission learning, vertebrates such as dogs (Cole and Wahlsten, 1968), mice (Kamin, 1957; Avcu et al., 2014), and fish (Woodard and Bitterman, 1973; Abramson et al., 1988; Portavella, 2004; Vindas et al., 2014) have demonstrated the ability to learn from omission. By omission learning, I refer to the ability to learn an association based on a predicted event *not occurring*, as opposed to learning from a stimulus presentation or offset. In contrast to vertebrates, invertebrates seem to fail on such omission learning studies, and only learn from stimulus offsets (Abramson et al., 1988; Wenner and Wells, 1990; Sanderson et al., 2013; Abramson and Wells, 2018).

#### Evidence That Vicarious Trial and Error, Episodic Memory, and Counterfactual Learning Emerged in Early Mammals

VTE is a behavior whereby an animal pauses at choice points and toggles its head back and forth, and “plays out” each option vicariously (reviewed in Redish, 2016). Recording studies have corroborated the hypothesis that animals are playing out these options vicariously, hippocampal place cells are shown to vicariously encode place sequences of each possible path (Johnson and Redish, 2007; Gupta et al., 2012). VTE has been shown across rodents, nonhuman primates, and humans (reviewed in Redish, 2016). Further, uniquely mammalian structures, such as the prefrontal cortex, are highly implicated in such VTE behavior. In contrast, I am not aware of any studies that have shown VTE behavior in non-mammals. Taken together, this is suggestive that VTE emerged in early mammals.

Counterfactual learning is when an animal learns from an alternative choice they *could* have made but did not actually make. Learning from counterfactuals has been shown in rodents (Lewis, 2014; Steiner and Redish, 2014), nonhuman primates (Abe and Lee, 2011), and humans (Zhang et al., 2015). Further, uniquely mammalian structures, such as the orbitofrontal cortex, are highly implicated in counterfactual learning (Gilovich and Medvec, 1995; Camille et al., 2004; Coricelli et al., 2005, 2007). In contrast, I am not aware of any studies that have demonstrated counterfactual learning in non-mammals. Taken together, this is suggestive that counterfactual learning emerged in early mammals.

A key test of the ability to engage in episodic memory is whether an animal can answer an unexpected Question about its own past. The ability to answer such unexpected Questions has been shown in mammals including dogs (Fugazza et al., 2020), rats (Crystal, 2013), and nonhuman primates (Menzel, 1999). The neural substrate of such episodic memory seems to be the hippocampus reactivating distributed representations across the neocortex (Eichenbaum et al., 2007). Such episodic memory has also been shown in pigeons (Zentall et al., 2008) and cephalopods (Billard, 2020). However, birds are poor model organisms for the amniote common ancestor, and cephalopods are a poor model organism for the bilaterian common ancestor; both seem to have independently evolved many unique brain structures (Roth, 2015). I am not aware of any tests of episodic memory, whereby animals answer unexpected Questions, in amniotes outside of birds and mammals. Taken together, this is suggestive, but far from conclusive, that this type of episodic memory emerged in early mammals.

One might think that vicarious trial and error is a requirement in order for “spatial maps” to be used by animals for map-based navigation, but this seems to not be the case. For example, fish do not show VTE behavior, but can remember locations in three-dimensional space (Karnik and Gerlai, 2012; Burt de Perera et al., 2016; Lucon-Xiccato and Bisazza, 2017; Wallach et al., 2018), and can generate novel paths to the goal, even if it requires losing sight of that goal and swimming further away from it at first (Gómez-Laplaza and Gerlai, 2010). This demonstrates some ability to generate spatial maps, some lightweight “working memory” (staying on task even though losing sight of a goal), and object permanence (Sovrano et al., 2018).

If much of the presumably intelligence spatial navigation tasks can be performed by animals without VTE, then what is the benefit of VTE? Some suggestive evidence can be seen in the superior performance of mammals on certain tasks requiring “hard choices.” For example, mammals tend to substantially outperform non-mammal vertebrates on detour tasks, where an animal has to make a roundabout path around a barrier in order to get to a goal (MacLean et al., 2014; Gatto et al., 2018; Macario et al., 2020). Mammals also seem to outperform non-mammals in delayed gratification tasks, whereby they have to resist choosing an immediate small reward in order to get a delayed larger reward (Stevens et al., 2010). Some evidence also suggests that early diverging vertebrates such as fish cannot learn to zero-shot update the reward value of places (Beyiuc, 1938).

#### Evidence That Anticipating Future Needs, Theory of Mind, and Learning Through Observation Emerged in Early Primates

The Bischof-Kohler hypothesis states that only humans can take actions to alleviate a need that they *will* have in the future, but do not currently feel (Bischof-Köhler, 1985). However, evidence now suggests that in addition to humans, many nonhuman primates are also capable of this ability (McKenzie et al., 2004; Mulcahy and Call, 2006; Naqshbandi and Roberts, 2006; Janmaat et al., 2014). In contrast, non-primate mammals such as rodents have been shown to be unable to anticipate future needs (Naqshbandi and Roberts, 2006). Consistent with this, the substrates of the ability to anticipate future need states and use them to change current decisions seem to be uniquely primate structures, such as the dorsolateral prefrontal cortex (McClure et al., 2004; Kim et al., 2008; Hare et al., 2009). This is suggestive that the ability to perform this task emerged in early primates.

Theory of Mind (ToM) refers to the ability to take the perspective of others and understand their intentions and knowledge. It is still controversial whether any animals outside of humans contain this ability. But the balance of evidence seems to favor the idea that many primates indeed have ToM, even if it is not as robust as that of humans. Diverse species of nonhuman primates pass “false belief tests” (Bräuer, 2014; Krupenye et al., 2016; Smith, 2016; Kano et al., 2019). Further, nonhuman primates can distinguish between accidental and intentional actions and can distinguish between someone “unwilling” to do an action and someone “unable” to do an action (Call and Tomasello, 1998; Tomasello et al., 2003; Call et al., 2004; Tomasello et al., 2005). Further, the two neural structures most implicated in the theory of mind, the superior temporal sulcus and the temporoparietal junction, seem to have uniquely emerged in early primates (Kaas, 2009). In contrast, the bulk of studies on non-primate mammals conclude that they do not have ToM (Byrne et al., 2001; Bräuer, 2014; Aldhous, 2015). There is some evidence of ToM in birds (Bugnyar et al., 2016), but as we have discussed, birds are a poor model organism for the amniote common ancestor. Taken together, this is suggestive that theory of mind, even in a primitive form, emerged in early primates.

Learning skills through observation has been demonstrated across diverse species of nonhuman primates (Tomasello et al., 1987; Meunier et al., 2007; Ferrucci et al., 2019). The neural substrate of learning through observation also seems to be uniquely primate structures, including the STS (Perrett et al., 1985; Puce and Perrett, 2003). A key feature of learning skills through observation in primates is the ability to infer the intention of movement, and not simply mirroring the movement. Consistent with this, the mirror neurons in the premotor cortex of nonhuman primates have been found to be selective for abstract *goals* (Rizzolatti et al., 2001; Fogassi et al., 2005). There is some evidence that fish and reptiles can learn paths through observation (Brown, 2015; Lindeyer and Reader, 2010; Wilkinson et al., 2010). However, this knowledge seems isolated to observing *paths* and is not readily passed down in generations (Lindeyer and Reader, 2010). Taken together, this is suggestive that learning motor skills through observation emerged in early primates.

It should be noted that there are many highly intelligent social behaviors that are observed across vertebrates well outside of the primate taxa. Empathy behaviors are seen in mice (Hofer, 1996; Bartal et al., 2011; Mogil, 2012; Rennie et al., 2013). Play has been observed in reptiles (Kramer and Burghardt, 2010), birds (Fagen, 1981), and mammals (Wojciech, 2009). Jealousy and fairness preferences have been observed in mice (Douglas, 2012). Reciprocity has been observed in fish (Brandl and Bellwood, 2015) and in mice (Viana et al., 2010). Complex understanding and implementation of social hierarchies have been observed in fish (Whoriskey, 1991; Grosenick et al., 2007; Reebs, 2010) and mice (Haller and Kruk, 2006). Kin recognition has been observed even in fish (Fricke, 1974; Spence et al., 2008; Reebs, 2010; Spence, 2011), reptiles, and non-primate mammals (Brennan and Kendrick, 2006). And movement imitation has been observed in reptiles (University of Lincoln, 2014) and rats (Seyfarth and Cheney, 2013). And even gaze following has been seen in reptiles (Simpson and O’Hara, 2018).

But despite the impressive complexity of these social behaviors, none of these in fact require the theory of mind or learning through observation, and all of them can be explained by the valence and mapping functionality of the vertebrate brain. Take empathy, for example, it merely requires cues of satisfaction or dissatisfaction of conspecifics to be “hardwired” to elicit similar valence responses in an individual the same way that predator smells are hardwired to elicit fear. Consistent with this, “pain” neurons in rats that get activated when in pain, also get activated when they see the pain in others (Netherlands Institute for Neuroscience—KNAW, 2019). Kin recognition, social hierarchies, jealousy, and play can all be explained in the same way—through cue recognition and hardwired valence. Gaze following and movement imitation can be explained as merely an impressively complex reflex.

#### Evidence That Language and Music Emerged in Early Humans

The key features of human language that differentiates it from other forms of animal communication seems to be the ability to “name” objects and organize these names with “grammar” (Berwick and Chomsky, 2017; Terrace, 2019). Despite many attempts, non-human primates have been shown to be unable to learn such human-like language (Terrace et al., 1979; Thompson and Church, 1980). A key feature in the ontogenetic development of language learning in humans seems to be “joint attention,” whereby a child and a parent can jointly attend to the same object, enabling the process of “naming” (Carpenter and Call, 2013). Attempts to demonstrate joint attention in nonhuman primates have shown negative results (Warneken and Tomasello, 2006; Warneken et al., 2006).

Additionally, music seems to be a human universal, shown across all human cultures (Brown and Jordania, 2011). A key feature of music is the ability to engage in “beat-based timing,” whereby a human can tap to a beat. Despite this being trivial in humans, nonhuman primates cannot learn to synchronize taps with an auditory or visual metronome, even after a year of training (Zarco et al., 2009; Honing et al., 2012). Nonhuman primates also struggle with relative pitch perception more than humans do, struggling to generalize melodies to transpositions (Hulse et al., 1984; D’Amato, 1988; Ralston and Herman, 1995; Ghazanfar, 2002).

Taken together, this is suggestive that language with words and grammar, along with key features of music, emerged in early humans.

### Evidence for Proposed Function of Specific Brain Modifications

#### Evidence That the Brain of Early Bilaterians Contained Valence Neurons and Neuromodulators Which Together Enabled Taxis Navigation

Many of the sensory neurons within early diverging bilaterians such as *C. elegans* can be interpreted as valence neurons. Many sensory neurons in *C. elegans* are directly modulated by internal states and thereby seem to directly encode valence as opposed to raw sensory information: hunger peptides modulate responses of neurons sensitive to food smells, pain, and various aversive smells (Davis et al., 2017; Lau et al., 2017; Rhoades et al., 2019). Further, temperature-sensitive neurons do not actually reliably encode temperature, but rather activate when a temperature rises above a homeostatic baseline—in other words, it is encoding the “negative valence” of temperature, not temperature *per se* (Luo et al., 2014). Food smells that trigger approach when hungry often have no effect when *C. elegans* is satiated (Davis et al., 2017). In *C. elegans*, some cues like carbon dioxide, which can signal both food as well as predators, shift from attractive when hungry to aversive when well fed depending on hunger state (Rengarajan et al., 2019).

It is likely that the cnidarian-bilaterian common ancestor had neurons that also contained neuropeptides that signaled internal states (Jekely, 2013), but the use of these peptides for such coordinated navigational decisions (locally search area, or relocated entirely, or rest) seems to either be unique to or have been highly elaborated in Bilateria. In Cnidaria, such neuropeptides seem to have primarily modulated independent reflexes, such as the likelihood of nematocyst release (Kass-Simon and Pierobon, 2007) or the swallowing reflex (Barron et al., 2010).

In early bilaterians, neuromodulators seem to have played a large role in navigational decisions, especially when it came to persisting navigational states. For example, in *C. elegans*, dopamine seems to trigger *dwelling* behavior whereby an animal engages in local area restricted search (Sawin et al., 2000), while *octopamine* seems to trigger *roaming* behavior whereby an animal relocates entirely (Churgin et al., 2017). Throughout Bilateria, neuromodulators have a remarkably consistent template for triggering affective states (Fellous, 1999; Lövheim, 2012).

The neural mechanisms of associative learning are remarkably conserved across bilateria. Across bilateria, animals use cAMP as well as NMDA and AMPA receptors in learning processes (Kandel, 2001, 2006; Dubnau et al., 2002; Farooqui et al., 2003; Glanzman, 2010; Hawkins and Byrne, 2015). And further, “third factor” neuromodulators such as dopamine and serotonin are involved in gating both presynaptic and postsynaptic learning processes across both vertebrates and invertebrates including diverse invertebrates such as crickets (Hammer and Menzel, 1998; Farooqui et al., 2003; Vergoz et al., 2007), fruit flies (Burke et al., 2012; Liu et al., 2012), honeybees (Hammer and Menzel, 1998; Farooqui et al., 2003; Vergoz et al., 2007), and *C. elegans* (Kusayama and Watanabe, 2000; Qin and Wheeler, 2007).

Further consistent with shared mechanisms of learning and affect, even early diverging invertebrates, such as *C. elegans* and planarians, show addiction to similar chemicals that manipulate dopamine and other monoamines as vertebrates do (Kusayama and Watanabe, 2000; Barron et al., 2009, 2010; Devineni and Heberlein, 2009; Kaun et al., 2011; Søvik and Barron, 2013).

#### Evidence That the Pallial-BG-Tectal Network Performed the Function of Model-Free Reinforcement Learning in Early Vertebrates, and Was Applied to Enable Map-Based Navigation, Interval Timing, and Omission Learning

The proposed “breakthrough” of early vertebrates was the emergence of model-free RL of the kind which included spatial maps and sensitivity to interoceptive information. Studies of the function of the structures that emerged in early vertebrates corroborate this idea. Substantial evidence suggests that homologous regions of the pallium generate a spatial map across vertebrates. The ability to navigate spatial maps is abolished when hippocampus homologs (present in the pallium) are lesioned across many early diverging vertebrates (reviewed in Murray et al., 2018), including goldfish and turtles (López et al., 2003; Durán et al., 2010; Broglio et al., 2015). Further, border cells, head direction cells, place cells, and velocity cells have been observed in the hippocampus-like structures across vertebrates, including the lateral pallium of early diverging vertebrates such as teleost fish (Vinepinsky et al., 2018, 2020), which is what you would expect if the lateral pallium were the homolog of the hippocampus and the neural substrate of the “spatial map”. Further, lesions to hippocampal homologs that impair spatial navigation also impair time perception (Meck et al., 1984, 1987, 2012; Melgire et al., 2005; Balci et al., 2009; Yin and Meck, 2014; Lucon-Xiccato and Bisazza, 2017), consistent with the idea that the pallium incorporated representations of both time and space. Time cells have also been found in the hippocampal complex (Eichenbaum, 2014), and time has been shown to be represented in the hippocampus as a map, just as space is (Oprisan et al., 2018). The cerebellum, another uniquely vertebrate structure, is also shown to be critical to absolute timing tasks (reviewed in Breska and Ivry, 2016).

Temporal difference learning signals are also observed in common vertebrate structures such as midbrain dopamine neurons, lateral habenula, and the basal ganglia across vertebrates, including early diverging vertebrates such as teleost fish (Li, 2012; Cheng et al., 2014). Further, the specific circuitry of the basal ganglia, which is conserved even in the earliest diverging vertebrates such as the lamprey, has been shown to be entirely consistent with a class of model-free learning algorithms called “actor-critic” models (Grillner and Robertson, 2016).

The specific neural substrates of avoidance and omission learning also corroborate the idea that the pallial-BG-tectal implemented a model-free RL algorithm. In model-free RL, we would expect avoidance learning to work in the following way. When an animal experiences unexpected pain, there should be a decline in dopamine (negative reward prediction), and they are driven to escape via amygdala-brainstem circuitry. Whenever pain is *offset*, this should lead to rebound excitation of dopamine (positive reward prediction, or “relief”). If specific actions consistently precede the offset of pain, this dopamine burst will drive plasticity in striatal circuits, which learn the contingency between a CS predictive of pain and the dopamine reward of relief. If this happens enough times, the CS will cease to be scary (pavlovian responses fade) and will simply drive the avoidance behavior that has been reinforced. Such an interpretation of avoidance learning has been suggested by others (Oleson et al., 2012).

The evidence supports exactly the above mechanism for how avoidance learning works in vertebrates. First, as expected from the above model, it has been shown that the basal amygdala to basal ganglia circuitry is required for active avoidance but not escape (which requires a basal amygdala to central amygdala circuit; Bandura and Rosenthal, 1966; LeDoux et al., 2016). Second, it is shown that during initial learning dopamine declines in the striatum, but after avoidance is well learned, dopamine increases, and the increase in dopamine is predictive of the performance of avoidance (Oleson et al., 2012). Crucially, this dopamine does not increase in the striatum if the pain is always inescapable. It was even shown that stimulating dopamine neurons during aversive cue increases active avoidance performance (Wenzel et al., 2018) while inhibiting dopamine in the striatum prevents avoidance (Wenzel et al., 2018). Third, it has been shown that pain or other noxious stimuli offset drives dopamine bursts in the striatum (Navratilova et al., 2015). Fourth, it explains why pavlovian fear responses fade while avoidance can maintain itself (Annau and Kamin, 1961; Kamin et al., 1963; Blanchard and Blanchard, 1969; Starr and Mineka, 1977; Kapp et al., 1979; Mineka, 1979; Bolles and Fanselow, 1980). And fifth, it is shown that avoidance responses seem to show signs of being habitual (extensive avoidance training makes the amygdala not required for avoidance anymore (Lázaro-Muñoz et al., 2010), although still being necessary for the acquisition (Choi et al., 2010).

This interpretation of the pallial-BG-tectal system is consistent with others who similarly suggest these structures together implement a model-free learning algorithm (Joel et al., 2002; Stephenson-Jones et al., 2013; Grillner and Robertson, 2016).

#### Evidence That the Neocortex Served the Function of Enabling Internally Invoked Simulations in Early Mammals, Which Was Applied for VTE, Counterfactual Learning, and Episodic Memory

The specific proposed model of ACC-sensory function presented in this article is consistent with various observations. There is emerging consensus that the neocortex, especially the sensory cortex, implements some form of a generative model (reviewed in Kersten et al., 2004; Knill and Pouget, 2004; Parr and Friston, 2018). Consistent with such a generative model, imagining a stimulus activates the same exact representations in the sensory neocortex as the stimulus itself (O’Craven and Kanwisher, 2000; Doll et al., 2015; Pearson et al., 2015). Further, lesions to specific areas of the sensory cortex create impairments both in perception and imagination within the same modality (Bisiach and Luzzatti, 1978; Farah et al., 1992).

There is less consensus regarding the function of the prefrontal cortex, which is further confused by the fact that the nomenclature of prefrontal regions in rodents often confuses the homology of these regions between rodents and primates. Recent research suggests that PL, IL, cg1, and cg2 regions of the rat prefrontal cortex are all homologous with the anterior cingulate cortex and medial cingulate cortex of primates (Laubach et al., 2018; van Heukelum et al., 2020). Through this lens, emerging evidence is consistent with the proposal that the frontal cortex builds a model of intent, which is used to trigger internal simulations.

For example, lesion studies provide evidence that the prefrontal cortex triggers internally invoked simulations in rodents: frontal lesions create impairments in all three proposed forms of internally invoked simulations, including vicarious trial and error, episodic memory, and counterfactual learning. More specifically, rodents with lesioned or inactivated prefrontal areas reduce their vicarious trial and error behavior (Schmidt et al., 2019), no longer have goal representations in the hippocampus (Ito et al., 2015), and are impaired in episodic memory tasks (Frankland et al., 2004). Evidence also suggests rats with frontal lesions become impaired at causal reasoning tasks, consistent with an inability to engage in counterfactual learning (Jones et al., 2012). Frontal lesions also make rodents uniquely impaired at spatial navigation tasks that require preplanning (Granon and Poucet, 1995). Rodents with frontal lesions struggle to stay on task in an ongoing plan and often do actions out of sequence (Seamans et al., 1995). Lesions of some of these cingulate regions in rats impair their ability to incorporate “effort” into their decision-making, as if they are unable to actually imagine how hard taking an action would be (Walton et al., 2003; Schweimer et al., 2005; Hu et al., 2021). Disconnecting areas of the cingulate from the amygdala in rats has the same effect (Floresco and Ghods-Sharifi, 2006). Further, temporarily inhibiting the hippocampus during tests where mice are asked to recall episodic memories abolishes their ability to do so (Crystal, 2013), consistent with the model whereby the frontal cortex makes inquiries for an episodic memory through its projections to the hippocampus.

Recording studies within the prefrontal cortex of rats also show evidence of modeling “intent”. In complex tasks sequences, ensembles of neurons in the prefrontal areas of rats become highly selective for the specific places in the task sequence and reliably track progress towards imagined goals (Cowen and McNaughton, 2007; Fujisawa et al., 2008). Further, in working memory tasks when rats must do tasks from memory without the presence of any cues to follow, neurons in the prefrontal cortex show delay activity (Baeg et al., 2003). The observation that the prefrontal cortex of rodents, such as the ACC, becomes particularly activated by surprise (Bryden et al., 2011) and error (Totah et al., 2009) is consistent with the model whereby during hard choices, the ACC pauses behavior and triggers internally invoked simulations to play out possible futures and resolve any conflicts or errors.

Even primates, who have many more prefrontal regions, still have these more ancient agranular frontal regions such as the ACC, which is homologous to most of the prefrontal cortex of non-primate mammals (Laubach et al., 2018; van Heukelum et al., 2020) and share many of these functions. The ACC in humans also gets uniquely activated during “errors” (Dehaene et al., 1994) and conflict (Carter et al., 1998; Braver et al., 2001). The ACC in humans also seems to encode locations in task sequences (Koechlin et al., 2002). Single neuron recordings of ACC within nonhuman primates show single neuron level selectivity for different actions (Nakamura et al., 2005), as well as selectivity for the serial order of tasks irrelevant of the actual movements made (Procyk et al., 2000; Procyk and Joseph, 2001). In humans, hippocampal lesions also create severe impairments both in the ability to recall autobiographical events and imagining potential future events (Addis et al., 2007; Hassabis et al., 2007). ACC lesions in nonhuman primates also impair the ability to stay on task during delay periods (Rudebeck et al., 2014).

The supposed “default mode network,” consisting of the mPFC, ACC, hippocampus, and the posterior cingulate all become uniquely active both during the retrieval of autobiographical memories as well as during imagining potential futures (Hassabis and Maguire, 2009; Martin et al., 2011; Andrews-Hanna et al., 2014b). Although some areas of the default mode network in primates include uniquely primate areas (such as granular prefrontal cortex), the DMN in primates also includes more ancient areas such as the ACC, which is also part of the purported DMN in rodents (Lu et al., 2012; Stafford et al., 2014; Grandjean et al., 2020), suggestive that such a network was present even in the very early neocortex.

It is interesting to note how many behavioral abilities that are attributed to the neocortex are readily performed by many animals that do not have a neocortex. Take object recognition for example. Complex object recognition, even of human faces, has been shown across phyla, including fish (Newport et al., 2016; Schumacher et al., 2016). Object recognition that is insensitive to changes in rotation and transformation has also been shown across non-mammalian phyla, also including fish (Newport et al., 2018). Object identification despite occlusion, demonstrating inference, has also been shown across non-mammalian phyla, including fish (Sovrano and Bisazza, 2007). However, consistent with the proposed function of the neocortex, mammals seem to be unique in their use of “mental rotation” to solve object recognition tasks. Mental rotation has been suggested by studies in monkeys, humans, and sea lions (Mauck and Dehnhardt, 1997), and negative results have been found in pigeons (Hollard and Delius, 1982). This would again represent the usage of such “simulation” functionality of the neocortex.

If simulating is so adaptive, then why did it only evolve in mammals and not in non-mammal vertebrates? One possible explanation is the fact that early mammals were likely arboreal species (Fröbisch and Reisz, 2009). Navigating tree branches with far eyesight presents unique challenges and evolutionary pressures not previously experienced: namely, irreversible choices. As a small animal living in trees, they had to plan their route well in advance when navigating across branches. And it is likely they had to very regularly experience novel branches. This perhaps created pressure for “vicarious trial and error”. Consistent with this, computational models have found that the usefulness of “planning” is directly tied to visual range. Visual range in water is so poor that computational models suggest planning in water is barely useful at all (Mugan and MacIver, 2020). However, inconsistent with this proposal is the fact that there are plenty of invertebrate and reptilian arboreal animals without the neocortex. Another hypothesis, as suggested by Mugan and MacIver (2020), is that many intellectual abilities occurred only in mammals due to the unique evolution of endothermy, which enabled much faster neural processing than when ectothermic. The neocortical generative model perhaps came at a high computational cost, and as such, without endothermy, nonmammalian vertebrates were prevented from garnering this adaptation. Consistent with this, the non-mammal vertebrates that demonstrate the most impressive intellectual abilities, birds, also seem to have evolved both endothermy (Walter and Seebacher, 2009) and neocortical-like structures (Tosches, 2021) through their own convergent evolution.

#### Evidence That the gPFC-PSC Network Performed the Function of “Mentalizing” in Early Primates, and Was Similarly Applied for Anticipating Future Needs, Theory of Mind, and Learning Through Observation

When identifying the neural substrates of mentalizing, it is important to draw a distinction between *emotional contagion* (e.g., “emotional empathy”) and mentalizing (e.g., “theory of mind”; Shamay-Tsoory, 2011). Each is a different process, and evidence suggests they have separate neural substrates. Emotional contagion is the process of reflexively adopting the emotional state of a conspecific based on various cues that reveal their emotions. In contrast, mentalizing is the process of actively considering another’s perspective, knowledge, or emotions by imagining oneself in another’s shoes. Emotional contagion has been shown in non-primate mammals such as rodents (Langford, 2006) whereas, as discussed above, mentalizing seems to be unique to primates. Consistent with this, the key frontal substrate for emotional contagion seems to be the ACC, which is homologous to regions of the frontal cortex in early mammals. For example, certain areas of the ACC are activated both by one’s own experience of pain and by watching a conspecific experience pain (Derbyshire, 2000; Jackson et al., 2006). In contrast, the neocortical areas most commonly implicated in mentalizing and theory of mind are not the ACC but instead contained within the gPFC-PSC network. More specifically, metanalysis that has reviewed a multitude of imaging studies have primarily implicated the dmPFC (BA 8, 9), amPFC (BA 10), TPJ, and STS as areas that are uniquely activated by tasks that require the theory of mind (Carrington and Bailey, 2009; Van Overwalle and Baetens, 2009). The idea that emotional contagion evolved first, subserved by older frontal regions, and ToM then emerged later, subserved by newer primate frontal regions, has similarly been proposed by other (de Waal, 2008). Further consistent with this, damage to granular areas of mPFC leaves the ability to simulate past or future imagined scenes intact but impairs the ability to imagine *yourself* in that scene. In contrast, hippocampal lesions impair the ability to simulate complex scenes but leaves the ability to imagine *yourself* in those scenes intact (Kurczek et al., 2015).

Several studies have shown a linear relationship between the volume of orbital PFC, medial PFC, and social network size (Powell et al., 2010; Lewis et al., 2011; Powell et al., 2012). It has also been observed that gray matter specifically in mPFC increases when macaques move from smaller to larger social groups (Sallet et al., 2011). Further, one of the only frontal regions that is disproportionately larger in humans than other primates is BA 10 (Semendeferi et al., 2001). All of this is consistent with the concept that these areas of gPFC subserve mentalizing, and that mentalizing was a key feature in supporting larger social groups in early primates and even larger social groups in early humans.

The above evidence implicates only a relatively small portion of the granular prefrontal cortex (BA 8, 9, 10) is related to the process of mentalizing. This suggests that the general function of the granular prefrontal cortex is not mentalizing, but rather mentalizing is merely a function subserved by specific areas of the granular prefrontal cortex. However, a broader examination of the different types of tasks involved in mentalizing implicates a much broader suite of areas within gPFC. For example, within “ToM” it has been argued that there is a dissociation between “affective ToM” and “cognitive ToM” (Shamay-Tsoory and Aharon-Peretz, 2007). Affective ToM refers to the ability to take the emotional perspective of another, whereas cognitive ToM refers to the ability to take the physical sensory perspective of another. When examining the neural substrates of these different types of ToM, broader regions of gPFC get implicated in addition to just medial BA 8, 9, and 10. Specifically, evidence suggests that the vmPFC (BA 11, 12, 14) is specifically necessary for the affective aspects of ToM whereas dlPFC (BA 44, 45, 46) is specifically necessary for the cognitive aspects of ToM. vmPFC lesions selectively impair affective ToM and not cognitive ToM (Shamay-Tsoory and Aharon-Peretz, 2007)—although admittedly given that this was examined in humans, some of these lesions may have included areas of standard mentalizing mPFC (BA 8, 9, 10) as well as areas of the ACC (BA 24, 32). In contrast, dlPFC disruption seems to selectively impair cognitive ToM (visual perspective taking; Conson et al., 2015; Qureshi et al., 2020) but not affective ToM (Kalbe et al., 2010). Disrupting the right TPJ function also disrupts visual perspective taking, while disrupting dmPFC does not (Martin et al., 2020). Some of these dissociations may explain why there is evidence that some individuals with extensive lesions of ACC and mPFC can retain features of self-awareness (Philippi et al., 2012) and theory of mind (Bird et al., 2004).

Another dissociation may be in what specific type of inquiry an individual is making into another’s mind. fMRI evidence suggests that dmPFC and vlPFC get more activated when considering how to make an observed character feel better than when merely trying to identify the observed character’s emotion (Reniers et al., 2013). On the other hand, the same study found that the dlPFC gets more activated when a participant is asked to “imagine how *they* *themselves* would feel” in an observed character’s situation than when merely trying to identify the observed character’s emotion.

There may be even more nuanced dissociations between the roles of various areas of the granular prefrontal cortex and various features of mentalizing. When comparing activation during first-person perspective taking to third-person perspective taking, areas of vlPFC (BA 44) and premotor cortex (BA 6) seem to be uniquely implicated in third-person perspective taking (David et al., 2006). Further, fMRI evidence suggests that amPFC (BA 10) builds models of person-specific theory of mind, while dmPFC (BA 8 and 9) is used for general processes of the theory of mind (Welborn and Lieberman, 2015). Other fMRI evidence suggests that amPFC is activated in any self-referential thinking, and only selectively during the considering of one’s own *mental state* (or presumably someone elses’), does dmPFC also become activated (Andrews-Hanna et al., 2010, 2014a). Left dlPFC may be specific for considering the mental state of others, while right dlPFC may be specific for considering the mental state of yourself (Otsuka et al., 2011). Yet another dissociation that has been proposed is that the TPJ is responsible for making mental inferences about others (such as about goals or beliefs), while the mPFC is responsible for attributing overall personality traits about yourself and others (Van Overwalle and Baetens, 2009).

Consistent with the idea that mentalizing about *yourself* is repurposed for the task of mentalizing about others, these same areas of the granular prefrontal cortex also get activated when considering your own mind state, including dmPFC (Gusnard et al., 2001; Gallagher and Frith, 2003; Amodio and Frith, 2006; Gilbert et al., 2006; Ochsner, 2008; Van Overwalle, 2009; Bzdok et al., 2012; Moran et al., 2012), amPFC/vmPFC (Lane et al., 1997; Phan et al., 2004; Lotze et al., 2007), dlPFC (Otsuka et al., 2011), and TPJ (Kelly et al., 2014). The general idea that there are common substrates, especially in mPFC, for imagining yourself in a future or past situation as well as for imagining being in the mind of another has been reviewed elsewhere (Buckner and Carroll, 2007; Jenkins and Mitchell, 2011). When evaluating your own personality traits or receiving evaluations of yourself by others, the same mentalizing network in mPFC activates (Ochsner et al., 2005; Gilbert et al., 2006). Further consistent with this idea that theory of mind of others is “bootstrapped” on a generative model of yourself, is the fact that the concept of “self” emerges first in child development before the theory of mind emerges (Rochat, 1998; Ritblatt, 2000; Keenan et al., 2005).

The argument in this article is that the overall function of mentalizing was not only applied to understanding the cognitive and emotional states of yourself and others (“theory of mind”), but also for anticipating future needs. The idea being that mentalizing about yourself in the future is the fundamental mechanism by which you can anticipate what your intentions and desires will be in that future situation, and thereby generate behaviors necessary for fulfilling those needs. A multitude of findings are consistent with the idea that the areas of the gPFC-PSC network specific for the *cognitive* theory of mind, such as the dlPFC, seem to also be key substrates for anticipating these future need states. Damage to the dorsolateral prefrontal cortex (dlPFC) dramatically impairs performance on tasks requiring abstract goal representations such A-not-B tasks (Diamond and Goldman-Rakic, 1989), consistent with the idea that dlPFC is the neural substrate of such goal representations and that it cascades these goals down the motor hierarchy where representations are progressively more “habitual”. dlPFC uniquely activates when considering future rewards but not present rewards (McClure, 2004; Tanaka et al., 2004; Berns et al., 2007; McClure et al., 2007; Kim et al., 2008). dlPFC is selectively activated when choosing a delayed reward over an immediate reward (McClure et al., 2004, 2007; Weber and Huettel, 2008). It is activated when avoiding temptation such as when dieters exert self-control (Hare et al., 2009). Damage to dlPFC leads to impairment in the ability to give up immediate rewards for future ones (Figner et al., 2010). And temporary inactivation of the dlPFC impairs people’s ability to forgo an exciting high-risk high reward option for a lower-reward lower-risk option (Knoch et al., 2006). Evidence also suggests that some types of “rule-based” categorization are unique to primates—consistent with the idea that gPFC enables the presentation of abstract goals and intentions (Soto and Wasserman, 2014).

This article also argues that the function of mentalizing in the gPFC-PSC network was also applied to enable learning through observation. There are several pieces of evidence for this. The same neurons in the premotor cortex activate for the “intentions” within yourself as when observing those same intentions in others (Amodio and Frith, 2006)—these have been called “mirror neurons.” Further, such mirror neurons have been identified in nonhuman primates (Gallese et al., 1996; Rizzolatti et al., 1996; Fogassi et al., 2005) while I am not aware of reports of mirror neurons in non-primate mammals. Although there is still controversy surrounding whether these mirror neurons are in fact modeling others’ movements (Hickok, 2009; Churchland, 2011).

Taken together, the above evidence suggests that a key, if not the primary, function of the gPFC-PSC network was to enable mentalizing—defined as building a generative model of one’s own mind state, inclusive of intentions, emotions, and knowledge. And this function was thereby applied in numerous adaptive ways, such as modeling the mind of others (theory of mind), anticipating future needs, and learning through observation.

#### Evidence That Modifications to the Arcuate Fasciculus Enabled Both Language and Music in Early Humans

There are compelling parallels between the connectivity of the AF-BG network and modern machine learning language models. The most popular language models, which have been shown to be capable of remarkably flexible sentence production and completion, are long-short-term memory (LSTM) models. LSTM models have “working memory” gates whereby the model can learn to maintain “context” from past words and sentences to the current word production (Mikolov et al., 2015; Dennis Singh and Lee, 2017; Ororbia et al., 2017). This is similar to the function proposed by frontal-cortex-basal-ganglia loops (O’Reilly and Frank, 2006). Further, these networks are recursive, whereby the next word is a function of the previous word, similar to the extensive recursive connectivity of both posterior and frontal cortex. The unique lateralization of language and music relative to other neocortical functions may be a requirement for the rapid sequence prediction in language and melody production.

Consistent with this interpretation of the AF-BG network, the arcuate fasciculus, and the basal ganglia are both highly implicated in language and music. Damage to AF impairs fluency, comprehension, and verbal working memory (Binder and Desai, 2011; Schomers et al., 2017). The development of the AF is correlated to the development of language abilities in children (Friederici, 2011; Yeatman et al., 2011; Skeide et al., 2016; Goucha et al., 2017; Schomers et al., 2017). The strength of connections in AF is correlated with word learning performance (Lopez-Barroso et al., 2013). It has long been known that language related tasks such as sentence generation activate and require a functioning Broca’s area, Wernick’s area, and the basal ganglia (Brown et al., 2006). However, less appreciated is the fact that music tasks such as melody generation activate the exact same network (Koelsch et al., 2002; Brown et al., 2006), although with a bias towards the right hemisphere, while language is biased towards the left hemisphere (Sammler et al., 2015). Consistent with a common neural implementation, children with language impairments also show musical impairments (Jentschke et al., 2008).

The direct projection from the motor cortex to laryngeal motoneurons, which is not found in nonhuman primates, is clearly the projection by which humans can control speech sounds (Simonyan and Horwitz, 2011). However, this was likely an addition after language had already emerged (Hewes et al., 1973; Corballis, 2002)—and not the fundamental modification that enabled language. Evidence for this can be seen in the fact that complex gestural languages (with words and grammar) are seen in deaf communities all over the world, none of which requires these descending laryngeal projections. Further, the neural substrates of such gestural language are highly overlapping with those of vocal-verbal language, implying a shared circuitry for “language,” independent of the modality (Neville et al., 1997). As such, I hypothesize that the big breakthrough that unlocked music and language was in fact the AF-BG network, and direct control of laryngeal motor neurons came later, enabling verbal language to replace the shorter-range gestural language.

## Discussion

Here I have argued that the proposed ordered set of five breakthroughs provides a first approximate explanation of a diverse set of both brain and behavioral modifications through major milestones in human brain evolution. This model of brain evolution provides a useful simplification through which to interpret brain modifications and the progressive complexification of behavior through phylogenetic refinement (Cisek, 2019).

### Caveats

By summarizing such a long history into only a handful of “breakthroughs,” I am undeniably simplifying the actual story. The objective is to provide a view of the “forest” of evolution at the cost of describing “the trees”. This approach will inevitably miss some important changes in brains and behavior. However, as argued above, a surprisingly broad set of brain structures and behaviors can be understood through a remarkably small number of “breakthroughs.” Perhaps this is not so surprising, given that perhaps brain evolution often occurred in fits and starts, where some adaptive structure was stumbled upon, rapidly elaborated on, and then brains remained relatively stable for a long period of time afterward. This can be seen simply in the transition from our primate ancestors to homo sapiens. For 30 million years primate brains remained mostly unchanged. And then, over the last 1 million years our ancestors’ brains expanded by a multiple of three. A million years ago humans could not speak flexible language, and 100,000 years ago they could—this is a split second from an evolutionary perspective.

It should also be noted that there is scant research with identically designed behavioral tests of animals across different phyla. This makes it both challenging and perilous to compare behavioral abilities across distant species. Many of the behavioral studies cited were not identically designed for each species tested nor evaluated using the same methods for different species, and hence such results must be interpreted with caution. For example, the experimental designs of asking a mouse to answer “unexpected Questions” from its own past are of course subtly different from asking the same Question of a cephalopod, who has a different sensory repertoire and is inevitably asked the Question in a different environment. As such, differences in results may be a consequence of the experimental design as opposed to differences in abilities. Further, comparative behavior research likely contains a positive results bias—whereby negative findings in animals are reported substantially less frequently than positive results (Fanelli, 2011). As such, there are scant reports of negative findings in general, and it is challenging to conclude the lack of ability in an extant animal simply because of a lack of reports of its presence—the absence of evidence is not the evidence of absence. As such, many pieces of this model are currently speculative and will require further studies comparing behavioral abilities across taxa.

### Comparison to Other Work

This work is highly inspired by others who have proposed ordered modifications in the evolution of brains: Eva Jablonka and Simona Ginsburg’s retelling of the evolution of learning systems (Vredeveldt et al., 2011), Paul Cisek’s theory of phylogenetic refinement (Cisek, 2019), and Elisabeth Murray, Steven Wise, and Kim Garham’s work on the evolution of memory systems (Murray et al., 2017). All three of these take an evolutionary approach to understanding how brains work today, by virtue of retelling the evolutionary steps by which they came to be.

My goal in this article is to add to this corpus of work in four ways. First, to provide an initial template and “first approximation” of the *entire* evolutionary story of the human brain, from the first bilaterains to the first homo sapiens. Second, to simultaneously hypothesize the emergence of both brain regions and adaptive behaviors that these brain structures enabled. Third, to directly incorporate dependencies between each sequence of changes. And fourth, to attempt to explain broad behaviors using common neural innovations, as opposed to specifically focusing on a type of behavior (such as learning or memory). In this last sense, this article is in some ways an attempt to bring together the ideas of Jablonka and Ginsburg, Cisek, and Murray, Wise, and Garham with the evolutionary neuroscience work of specific brain structures from Kaas (2009) and Striedter and Northcutt (2020).

The most well-known model of brain evolution is Paul MacLean’s Triune Brain (MacLean, 1990), where he named the three brain systems that he argued evolved sequentially: the “Reptilian Complex,” the “Paleomammalian Complex,” and the “Neomammalian Complex”. Despite how well known the model is, it has been highly discredited and demonstrated to be completely wrong (Cesario et al., 2020). A key flaw in MacLean’s model is the conceptualization of extant animals such as reptiles or monkeys as somehow “lower” animals, and humans as “higher.” His idea that the human brain has a “reptile brain” and a “monkey brain” within it, incorrectly conceptualizes an extant monkey brain as being more primitive than a human brain. The truth is of course that all extant animals have gone through evolution for the same amount of time and evolved from common ancestors. In contrast, the model of five breakthroughs proposed here is crafted to discuss specifically the evolutionary process only within the human lineage and is not meant to be used to make comparisons to extant animals today.

## Data Availability Statement

The original contributions presented in the study are included in the article, further inquiries can be directed to the corresponding author.

## Author Contributions

MB conceived the overall theory and wrote the entire manuscript.

## Conflict of Interest

The author declares that the research was conducted in the absence of any commercial or financial relationships that could be construed as a potential conflict of interest.

## Publisher’s Note

All claims expressed in this article are solely those of the authors and do not necessarily represent those of their affiliated organizations, or those of the publisher, the editors and the reviewers. Any product that may be evaluated in this article, or claim that may be made by its manufacturer, is not guaranteed or endorsed by the publisher.
